# Enhanced PRIM recognition using PRI sound and deep learning techniques

**DOI:** 10.1371/journal.pone.0298373

**Published:** 2024-05-01

**Authors:** Seyed Majid Hasani Azhdari, Azar Mahmoodzadeh, Mohammad Khishe, Hamed Agahi

**Affiliations:** 1 Department of Electrical Engineering, Shiraz Branch, Islamic Azad University, Shiraz, Iran; 2 Department of Electrical Engineering, Imam Khomeini Marine Science University, Nowshahr, Iran; Karunya Institute of Technology and Sciences, INDIA

## Abstract

Pulse repetition interval modulation (PRIM) is integral to radar identification in modern electronic support measure (ESM) and electronic intelligence (ELINT) systems. Various distortions, including missing pulses, spurious pulses, unintended jitters, and noise from radar antenna scans, often hinder the accurate recognition of PRIM. This research introduces a novel three-stage approach for PRIM recognition, emphasizing the innovative use of PRI sound. A transfer learning-aided deep convolutional neural network (DCNN) is initially used for feature extraction. This is followed by an extreme learning machine (ELM) for real-time PRIM classification. Finally, a gray wolf optimizer (GWO) refines the network’s robustness. To evaluate the proposed method, we develop a real experimental dataset consisting of sound of six common PRI patterns. We utilized eight pre-trained DCNN architectures for evaluation, with VGG16 and ResNet50V2 notably achieving recognition accuracies of 97.53% and 96.92%. Integrating ELM and GWO further optimized the accuracy rates to 98.80% and 97.58. This research advances radar identification by offering an enhanced method for PRIM recognition, emphasizing the potential of PRI sound to address real-world distortions in ESM and ELINT systems.

## 1. Introduction

The topic of automation holds significant importance in contemporary ELINT and ESM systems [1]. The increasing intricacy of electronic warfare (EW) situations is the underlying cause. In order to achieve this objective, it is necessary to de-interleave the interleaved pulses emitted by various radars. Subsequently, each radar signal must be accurately identified and subjected to analysis in an automated manner without any manual intervention.

PRIMs are a significant component of radar signal analysis since they offer crucial insights into the origin of radiation and possible hazards [2–4]. PRIMs play a pivotal role in signal processing and serve as a fundamental point of reference for discerning the origin of radiation inside such systems [2]. Furthermore, radar warning receivers (RWR) and jammers employ pulse repetition interval (PRI) characteristics [5, 6].

**Fig 1:**
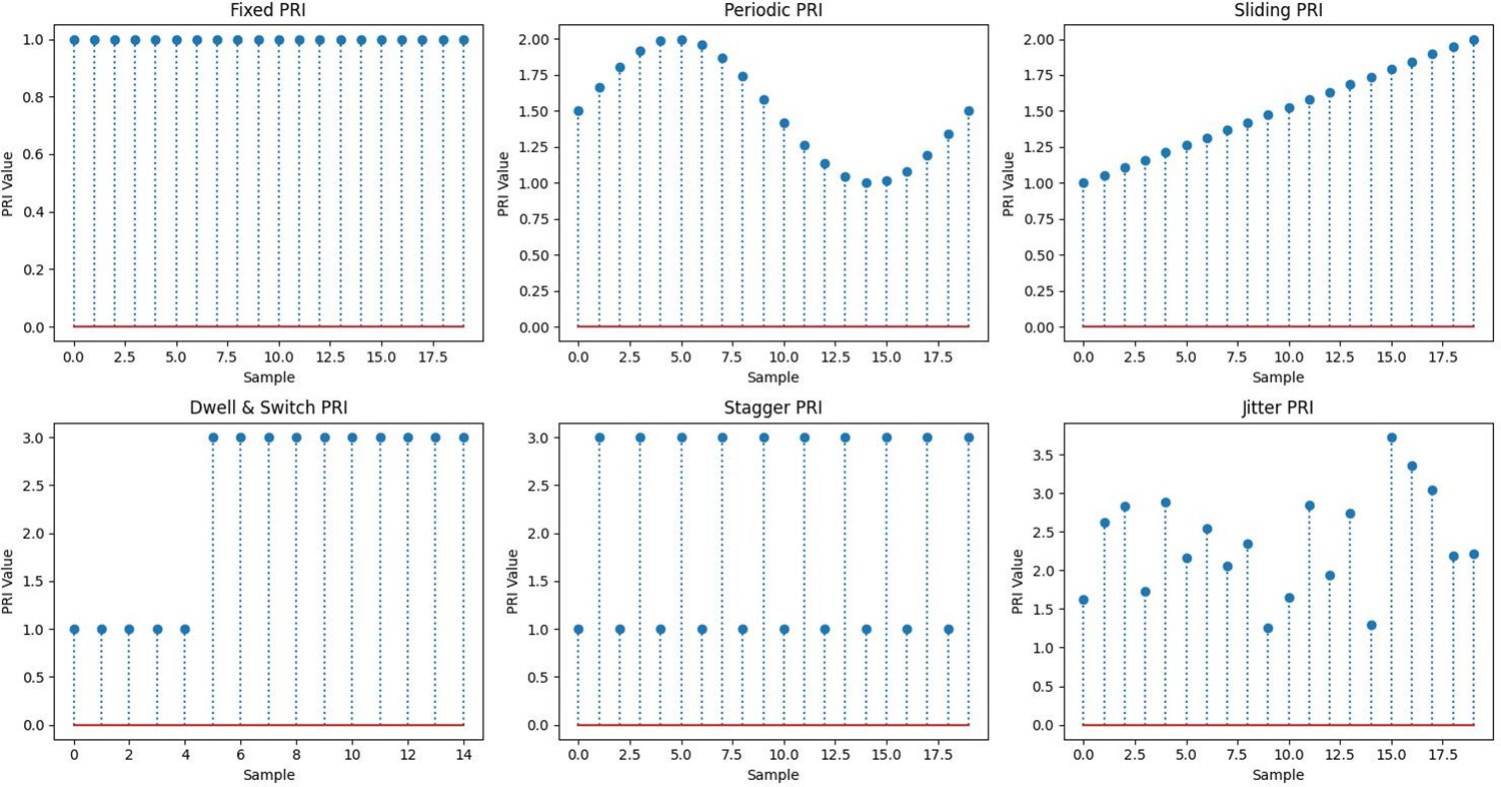
Illustration of various pulse repetition interval modulations [2].

PRIM is a significant challenge in radar signal processing inside ELINT and ESM systems. In the field of radar technology, it is commonly observed that there are six primary types of PRIM techniques employed. These techniques include simple, stagger, jitter, dwell and switch (D&S), periodic, and sliding modulations [6]. Fig 1 depicts different forms of PRIMs, visually representing the variety in modulation types and their respective patterns [2].

Table 1 lists several common variations of PRI, as referenced in the identified source, providing detailed insights into each type’s unique characteristics and specifications. The primary objective of the ESM and ELINT analyst is to classify radar emitters by analyzing changes in PRI, which is contingent upon specific PRI variations associated with each category [6].

**Table 1:** Common variations of pulse repetition interval modulations [6].

Type	Typical Function	Remarks
**Constant**	Search or track radars are commonly used in various applications.	The magnitude of variations in the average PRI value is often below 1%. MTI and pulse Doppler systems are commonly connected with highly stable constant PRIs.
**Jittered**	Mitigates the impact of certain forms of interference and jamming.	Significant fluctuations amounting to approximately 30% of the mean PRI value.
**Dwell & switch**	One of the critical challenges in pulse-Doppler radar systems is the presence of velocity ambiguities, which can affect the accurate determination of velocity or range.	The bursts of pulses exhibit a phenomenon wherein many stable Pulse Repetition Intervals (PRIs) are alternated between consecutive bursts.
**Stagger**	The objective is to reduce the presence of blind rates in Moving target indicator platforms.	A periodic pattern was observed in which many stable PRIs were switched occurs on a pulse-to-pulse cycle.
**Sliding**	To ensure continuous height covering in elevation scans or to prevent eclipse, it is necessary to maintain a consistent level of height observation.	Typically, the max Pulse Repetition Interval observed at the slightest elevation is generally found to be less than six times the min Pulse Repetition Interval watched at the top peak.
**Scheduled**	Electronic scanning employs computer-controlled systems that provide many features, including but not limited to search and tracking.	Many intricate patterns, including periodic ones, can adjust and conform to specific circumstances.
**Periodic variations**	The topic under consideration pertains to guiding missiles, explicitly focusing on avoiding eclipsing and range.	The observed fluctuations in the average PRI exhibit nearly sinusoidal patterns, with amplitudes reaching up to 5%. Rates of greater than 50 Hz or higher
**Pulse groups**	There is a potential for enhancing either the range or speed accuracy.	Additionally, this technology is employed for the Identification of Friend or Foe (IFF) and beacon interrogation.

Researchers have achieved notable advancements in PRIM recognition in the past several years by developing algorithms and methodologies [7, 8]. Recognition of diverse PRIM signals presents considerable difficulties owing to their intricate waveforms and the diversity observed in operational circumstances. The limitations of conventional signal processing techniques sometimes need to be revised to ensure the accuracy of detecting and identifying PRIMs. The primary constraints mainly arise from the challenges of developing algorithms capable of effectively managing the diverse range of PRIM variables, including pulse repetition frequency (PRF), pulse width (PW), and modulation methods. In addition, the intricate nature and various characteristics of PRIMs provide considerable obstacles in advancing machine learning (ML) methods designed for PRIM identification. A significant challenge during ML methods requires much training data. This task proves arduous in numerous real-world scenarios. Another obstacle is developing algorithms that exhibit resilience due to alterations in PRIM variables, such as PRF fluctuations or signal to noise ratio (SNR) variations. Resolving these issues is essential in advancing precise and resilient PRIM recognition techniques for contemporary radar systems employing a diverse array of PRIMs [9].

Hence, the objective of this investigation is to make a scholarly contribution to the domain of radar signal processing, an innovative methodology for identifying and classifying various types of PRIMs inside radar signals that are contaminated by noise. This study employs a deep learning-based approach to effectively detect PRIMs by utilizing the capabilities of DCNNs [10].

This study suggests using an ELM instead of the fully connected layer to provide a real-time processor [11–13]. Utilizing a DCNN automated feature, in conjunction with ELMs, can effectively tackle the problems associated with manual feature extraction and the elongation of training time in the proposed two-phase technique.

The Random Vector Functional Link (RVFL) [14] is utilized to establish the foundation of the ELM, resulting in a highly efficient and adaptable system [12, 13]. Research shows that engineering applications use ELM regularly [15–17]. However, it should be noted that there are indeed obstacles associated with ELM [18, 19], such as the need for many hidden nodes to provide better generalization and the requirement to choose appropriate activation functions.

ELMs, in contrast, strive to minimize training errors and ascertain the minimum norm of output weights. To mitigate the impact of poorly conditioned matrices on the accuracy of results, the input weights and biases employed in ELM are selected randomly. Consequently, the resulting matrix may not accurately represent the total column rank [17, 20, 21]. Therefore, this work utilizes a GWO algorithm to improve ELM’s conditioning and ensure optimal solutions are attained. This research suggests replacing the final conventional fully connected layer in DCNN with ELM for real-time training and testing purposes. A GWO algorithm is presented as a solution to the issues of ill-conditioning and inconsistency faced by the classical ELM. The proposed technique aims to provide real-time structure and high-accuracy detection.

GWO is a leading optimization application due to its excellent performance and flexibility. GWO was founded by examining the predatory actions of grey wolves [21, 22]. The concept is straightforward and can be easily implemented with little lines of code, making it accessible to many users. GWO has superior robustness in parameter regulation compared to other evolutionary algorithms, resulting in enhanced computing efficiency [21, 23]. Therefore, this work aims to utilize GWO as an alternative optimization technique for the ELM and integrate it into the PRIM system.

The methodology employed in the present investigation is grounded on prior scholarly investigations. It seeks to address many noteworthy issues, such as the presence of missing and spurious pulses and the vast array of characteristics exhibited by various PRIMs. The study’s experimental results demonstrate the effectiveness and robustness of our methodology, as evidenced by the achievement of significant levels of accuracy when applied to various radar signals.

### The primary outcomes of this study are as follows

1. The present study introduces a novel approach consisting of a three-phase methodology. This methodology utilizes a transfer learning-based DCNN as a feature extractor, an ELM for real-time recognition of the six often occurring forms of PRIM, and a GWO algorithm to improve the network’s resilience and stability.
2. This study introduces using PRI sound to identify its modulation for the first time. The utilized data sets consist of authentic data obtained through designing, constructing, and deploying the necessary system in a region characterized by a significant concentration of radar signals.
3. The efficacy of eight distinct benchmark transfers learning-based DCNNs is initially evaluated on the given dataset.
4. In addition, an examination and assessment of the efficacy of integrating eight variations of TDCNN with ELM are conducted on the given dataset.
5. In addition, the two transfer learning-based DCNN-ELM networks that yielded the most optimal outcomes on the dataset are also chosen and integrated with the GWO algorithm after scrutinizing and assessing the same dataset.
6. The results showed that VGG16 and ResNet50V2 models obtained the best recognition accuracy with values of 95.38% and training time of 38.92 seconds and 96.92% and training time of 442.75 seconds, respectively. These values increased to 98.46%, a training time of 60.97 seconds, and a 99.06% training time of 276.4 seconds with the evolution of these networks with ELM and GWO, respectively.

The structure of the paper is as follows: In Section 2, you will find a comprehensive overview of the literature currently available in the field. Section 3 critically examines the background knowledge relevant to the study. Section 4 provides an introduction to the hybrid model that is being proposed. Section 5 presents the simulation, outcomes, and discussion. Ultimately, the findings are briefly outlined in Section 6.

## 2. Literature review

The PRIM approach is a commonly utilized technique in radar relationships involving data modulation onto a radar signal’s PRI [24]. PRIM has garnered significant interest in contemporary times because it can deliver elevated data rates and ensure secure connection [25]. Nevertheless, PRIM signals are susceptible to several external factors, such as interference, noise, and jamming, all of which can potentially impact the overall effectiveness and efficiency of PRIM-based systems [26].

The available methodologies can be broadly classified into four distinct categories: statistical-based approaches, decision tree-based approaches, histogram-based approaches, and learning-based approaches [9].

As referenced in [27–30], most collaborative techniques rely on histogram operations. In the context of these methodologies, establishing an appropriate threshold typically emerges as the foremost pivotal concern. Additionally, the quantity of pulses must be sufficiently large in order to generate a well-defined histogram. Additionally, it is essential to consider practical considerations. In a previous study [30], the authors simulated 5,000 pulses emitted by three low PRF radars. It is worth noting that such scenarios are infrequently encountered in contemporary EW settings, primarily due to the radar antenna scan and the deficient side lobe levels.

Furthermore, it is imperative to consider the detrimental consequences that arise from the absence and erroneous occurrence of pulses during the recognition process. These phenomena significantly impact the majority of histogram-based algorithms. Due to their inherent simplicity, these techniques are limited in their applicability to a select range of PRIM schemes and exhibit notable performance degradation in the presence of noise.

Additional approaches can be observed in the relevant scholarly works. In [31], the authors treat every time of arrival (TOA) as an individual observation and each emitter as a distinct target. Utilizing a Kalman filter enables tracking individual emitter pulses and facilitates the prediction of forthcoming pulses. Despite the method’s capability to handle moderate levels of spurious and missing pulses, the simulated situation is limited in complexity as it encompasses only three forms of modulations: primary, jitter, and stagger.

Typically, scholars primarily studying PRIM recognition choose to employ feature-based methodologies. Like other classification issues, the initial phase in this process involves feature extraction. In this procedure, a limited to extensive number of characteristics are derived from an unadulterated signal, and those possessing the most excellent discriminatory capability are employed. Specific features include the capacity to effectively distinguish a single category from the rest, whereas others exhibit the ability to discern distinct groups of data accurately. In the methods described in [32, 33], a collection of features is extracted through autocorrelation. However, Reference [34] employs five distinct parts, three of which exclusively differentiate a particular form of PRIM, while the remaining two features discriminate between the other types.

The analysis of PRI is conducted in [35] through the utilization of the decimated walsh-hadamard transform (WHT). The approach employed in this study is a threshold-based strategy, wherein only three specific modulation types are considered: simple, jitter, and stagger. These modulation types are further categorized into up to four levels. Further examination of the identification of various kinds of PRIs and the detrimental consequences of the absence and erroneous presence of pulses may be found in [36]. This study employs a hierarchical approach, incorporating features based on wavelet analysis and intuitive features. The works cited [37–40] also show the utilization of intuitive characteristics.

Another significant aspect of this framework is the classification methodology. Decision Tree has been employed as the classifier in various investigations, including those referenced [35, 40] One of the primary limitations of decision-tree-based methodologies is the requirement for manually determined thresholds. This process is not only time-consuming but also highly susceptible to variations in noise levels and changes in PRI parameters [9].

The authors of [41] propose the utilization of a neural network classifier featuring a solitary hidden layer. This classifier is developed using a dataset including second differences in TOA arrival times. The authors of [37] present a feed-forward neural network architecture with an input layer with three distinct characteristics and a solitary hidden layer containing eight neurons. This methodology needs to be revised in categorizing and identifying only four different patterns of PRI change.

Acknowledging that the aforementioned learning-based strategies necessitate a comprehensive feature design and extraction procedure before utilizing the neural network is essential. This limitation hinders the approaches’ ability to adjust to variations in the pattern of PRI changes rapidly. Nevertheless, one notable benefit of intelligent techniques is their ability to identify several fundamental ways of PRI changes using different methodologies. Nevertheless, the drawbacks are comparable as they entail a substantial workload in data preprocessing and an inability to effectively adjust to environments characterized by a significant prevalence of missing and false pulses.

Deep learning (DL) has recently gained prominence as a formidable tool in various classification endeavors [42–44]. Notably, researchers have explored the application of DL in radar signal recognition. This is due to the inherent capability of DL to autonomously extract signal characteristics, leading to significant achievements in this domain. Various disciplines have been extensively explored, including image processing, speech recognition, and object detection [45].

The authors introduced a consolidated approach, deep learning-based multitasking learning (DMTL), to perform the five PRIM recognition of radar pulses [3]. The simulation findings indicate that the precision and accuracy of modulation recognition is 73.2%. This assessment considers an equal rate of 30% for spurious and missing pulses. The dataset comprises 10,000 samples representing five different PRI modulation types.

The authors in [46] proposed an attention-based recognition framework, known as the recurrent neural network (ARNN), for classifying pulse streams into six types of PRIMs. This framework is designed to handle high proportions of missing and spurious pulses. The simulation results demonstrate that this model achieves a PRM recognition accuracy of 92.18% while using attention and 89.56% without attention. This study involves a dataset of 240,000 data samples, with a false pulse rate of 70% and a missing pulse rate of 50%.

Scholars have recently investigated applying DCNNs in PRIM recognition. CNNs have demonstrated considerable efficacy across various applications owing to their inherent capacity to autonomously acquire and discern characteristics from unprocessed data [47–49].

The authors of [50] suggested a method based on DCNNs for recognizing seven different patterns of PRIM. The simulation results indicate that the total recognition accuracy is 96.1%, with a maximum of 50% lost pulses and 20% spurious pulses. The dataset has 25,000 samples encompassing all PRIM types.

Reference [9] introduces a unique technique based on DL. This technique utilizes a DCNN to classify seven distinct patterns of PRI changes. The simulation findings indicate that the overall recognition accuracy is approximately 96%, whereas the rates of missing and spurious pulses randomly range from 25% to 30%. The dataset consists of 3,000 samples for each PRI modulation type.

The paper introduces a novel approach that utilizes the inherent characteristic of the temporal convolutional network (TCN) [2]. The simulation findings demonstrate that this method can accurately categorize seven distinct variations of PRI modulation, even in the presence of a higher proportion of missing and false pulses (up to 30%). The suggested model can effectively differentiate between seven forms of PRI modulation with an accuracy of over 98%. The results are derived from a sample size of 40,000 tests, chosen randomly from a pool of seven distinct modulations, each having an equal likelihood of being selected.

In a subsequent study, the authors [51] presented a DCNN system for PRIM classification. The simulation results demonstrate that this method can accurately classify eight distinct kinds of PRIM, achieving an overall recognition accuracy of 98.5%. This performance is achieved even when there is a 15% ratio of missing pulses and a 15% ratio of spurious pulses. The dataset consists of 16,000 samples for each PRI modulation type.

The findings of various methods utilizing DL for the recognition and classification of PRIMs are summarized in Table 2.

**Table 2:** The summarizes of the results of a number of DL methods.

Ref	Number of samples in the dataset	Model	Accuracy		Limitations			Number of PRIM Types
					Missing pulses	Spurious pulses	Outliers resulting from radar antenna scanning	
**[3]**	10,000	**DMTL**	73.2		30%	30%	Is not considered.	5
**[46]**	240,000	**ARNN**	With Attention	92.18%	Similar to the real data		Is not considered.	6
			Without Attention	89.56	70%	50%		
**[50]**	25,000	**DCNN**	96.1%		50%	20%	Is not considered.	7
**[9]**	21,000	**DCNN**	Less than 95%		20–30%	20–30%	Is not considered.	7
[2]	40,000	**TCN**	more than 98%		5–30%	5–30%	1–10%	7
[51]	128,000	**DCNN**	98.5		15%	15%	Is not considered.	8

One area for improvement is that existing techniques mainly rely on features, which may not fully capture PRIM patterns’ complex and diverse nature. Moreover, these methodologies may demonstrate a restricted level of resilience when confronted with noise and interference, potentially undermining the system’s overall effectiveness in practical situations. Hence, this research introduces a novel approach that employs a DCNN as a feature extractor, ELM for real-time identification of PRIM designs, and WGO to enhance the network’s robustness. The methodology presented in this study has been devised to tackle the inherent limitations of existing techniques. It aims to improve the accuracy and consistency of PRIM detection, particularly in the presence of noise and interference. In addition, the expansion of datasets to encompass a broader range of PRIM signals with varying SNRs could enhance the progress and evaluation of PRIM recognition techniques.

The DCNN algorithms do not incorporate preprocessing steps like signal preparation or feature extraction. The efficacy of DCNNs in PRIM recognition remains evident, even when confronted with significant instances of missing and spurious pulses. Nevertheless, it is crucial to acknowledge a significant detrimental impact overlooked in the analysis, namely the presence of substantial outliers resulting from radar antenna scanning [2].

All the methodologies above have employed simulated data throughout their training and evaluation processes. The training and evaluation processes for accurate data pose significant challenges and consume much time at each level. These challenges arise from the presence of missing pulses and unexpected spurious pulses. Consequently, users experience delays of several hours before receiving feedback on their selected model for the intended diagnosis. Moreover, it is essential to note that all of these methodologies necessitate the utilization of an extensive dataset across all training and evaluation phases. The proposed method incorporates using PRI sound from actual radar systems, a novel approach that has yet to be previously employed. This PRI sound is utilized throughout all training and evaluation phases, marking a significant advancement in the field.

## 3. Background knowledge

This part presents a comprehensive overview of the underlying fundamental principles and essential concepts about the PRI Sounds, CNNs, GWO, and ELM algorithms.

### 3.1 Pulse repetition interval sound

The historical technique of PRI analysis involves using a loudspeaker or headphones to perceive the pulse train’s sound audibly. This remains relevant and valuable in contemporary times. The significance of pulse stretch circuitry is underscored by the low-duty cycle shown by radar signals. Furthermore, constant amplitude pulses may be employed due to the potential confusion arising from wildly fluctuating amplitudes, as stated in reference [6].

One straightforward approach is concurrently monitoring an audio oscillator alongside the radar pulse sequence. The analyst aligns the tonal characteristics of the generator with those of the pulse train by detecting beats, similar to the process of tuning a musical instrument. Novice analysts may mistakenly configure the audio oscillator to a harmonic or subharmonic of the PRI. However, this error is infrequently seen once sufficient experience is gained. The analyzer gradually increases the sound volume until the rhythm note becomes audible. The beat note frequency is equivalent to the disparity between the audio oscillator frequency and the PRF, which can be calculated as the reciprocal of the PRI. The audio oscillator is adjusted by the analyst until the frequency of the beat note reaches zero, resulting in the disappearance of the beat. Under optimal conditions, the margin of error is around ±20 Hz, as this value represents the minimum threshold of human auditory perception. Scanning can provide additional mistakes that make perceiving the beat note more challenging [6].

Contemporary ELINT devices are engineered to generate auditory signals, even when the PRF exceeds the threshold of human auditory perception. The process involves the nonlinear mapping of the authentic PRF to generate a synthetic PRF sound. For instance, frequency ranges up to 1 kHz are faithfully replicated without alteration. According to [6], it is possible to map PRFs ranging from 1 to 200 kHz onto a narrower range of 1 to 20 kHz.

### 3.2 Convolutional neural networks

CNNs are deep neural networks that excel in image identification and classification. CNNs are specifically engineered to acquire spatial hierarchies of information autonomously and adaptively by utilizing the backpropagation algorithm. CNNs commonly comprise the subsequent layers [42]:

1. The input layer is designed to receive the image input.
2. The convolutional layer performs a convolution operation on the input and then passes the resulting output to the subsequent layer. This technique facilitates the network’s ability to concentrate on specific local locations and acquire diverse properties.
3. The activation layer is typically implemented after each convolutional layer. The model incorporates a non-linear activation function, such as a rectified linear unit (ReLU), which enables it to acquire knowledge from the error and adapt accordingly.
4. The pooling layer is positioned after the activation layer and conducts a down-sampling operation across the spatial dimensions. This process reduces computing complexity by decreasing input dimensionality. Max and Average Pooling are popular pooling layers in neural networks.
5. The wholly connected layer consists of neurons that establish connections with all activations in the preceding layer, similar to conventional neural networks. At a higher level of abstraction, they can be conceptualized as classifiers.
6. The output layer generates the ultimate output of the neural network.

Training a CNN entails utilizing labeled training data, wherein the weights and biases within the network are iteratively adjusted [52]. This process involves applying backpropagation to propagate errors from the output to the input layer [52]. Optimization algorithms like Gradient Descent are employed to optimize the network’s performance. CNNs are instrumental in various applications, including but not limited to Image and Video Recognition, Image Analysis, Autonomous Vehicles, Healthcare (e.g., Medical Image Analysis), and Natural Language Processing (when combined with other types of architectures)

Some of the renowned CNN architectures include LeNet-5 [53], AlexNet [54], ZFNet [55], GoogLeNet [56], VGGNet [57], ResNet {Szegedy, 2017 #57. each with its unique characteristics and enhancements over its predecessors.

#### 2.2.1 MobileNetV2

MobileNetV2 represents a notable advancement compared to its predecessor, MobileNetV1, tailored explicitly for utilization in mobile and edge computing devices. Inverted residuals and linear bottlenecks are employed to enhance the propagation of information and gradients within the network. The software possesses a low weight and high efficiency, rendering it appropriate for real-time applications on devices with limited resources {Gulzar, 2023 #58}.

#### 2.2.2 Xception

Xception can be regarded as an expansion of the Inception architecture. Using depthwise separable convolutions instead of conventional Inception modules enables the network to learn cross-channel and spatial correlations independently. This results in enhanced efficiency and performance [58].

#### 2.2.3 EfficientNetB0

EfficientNetB0 is the base model of the EfficientNet family, focusing on balancing accuracy and computational efficiency. A compound scaling method is used to optimize performance across different scales by uniformly scaling the network’s depth, width, and resolution [59].

#### 2.2.4 EfficientNetV2B2

EfficientNetV2 represents an enhanced iteration of the existing EfficientNet models. This study aims to strengthen the optimization process’s precision and effectiveness. The approach employs a combination of compound scaling, model fusion, and progressive learning techniques to enhance performance while minimizing computational resources. The designation "B2" denotes a distinct variant or arrangement of the EfficientNetV2 concept [60].

#### 2.2.5 VGG16

The VGG16 model is a type of neural network architecture known as a DCNN. Researchers from the University of Oxford, K. Simonyan, and A. Zisserman created it. The design of the VGG16 model is notable for its focus on simplicity. It consists of multiple layers of 3x3 convolutional layers stacked on top of each other, with the depth of the layers increasing as you move further into the network. Once these convolutional layers have been processed, fully linked layers are utilized [61, 62].

#### 2.2.6 ResNet50V2

ResNet50V2 is an enhanced iteration of the initial ResNet50 architecture. Skip connections, shortcut connections, and bypass specific layers within the neural network architecture. This technique becomes beneficial in addressing the vanishing gradient problem, hence facilitating the successful training of deep networks. Including the "V2" designation signifies specific alterations and enhancements made to the initial ResNet framework [63, 64].

#### 2.2.7 MobileNetV3Small

MobileNetV3Small is another member of the MobileNet family, designed explicitly for resource-constrained environments. It incorporates advancements in architecture search and hardware-aware training, emphasizing efficiency and performance on mobile devices [65].

#### 2.2.8 DenseNet121

DenseNet121 belongs to the family of Densely Connected Convolutional Networks. The connectivity pattern of this network architecture establishes a feed-forward linkage between each layer, facilitating optimal transmission of information across all layers. The dense connection network of this approach allows for fewer parameters while achieving a high level of precision [66, 67].

### 3.3 Extreme learning machine

The ELM is a popular Single-hidden Layer Neural Networks (SLNN) learning algorithm. Its various versions are commonly employed in sequential, batch, and incremental learning due to their rapid and efficient learning speed, suitable generalization capability, fast convergence rate, and straightforward implementation [11]. In contrast to conventional learning algorithms, the fundamental objective of the ELM is to enhance generalization performance by minimizing the norm of the output weights and reducing the training error. According to Bartlett’s theory on feed-forward neural networks [21], networks with smaller weights examples will likely exhibit improved generalization performance.

**Fig 2:**
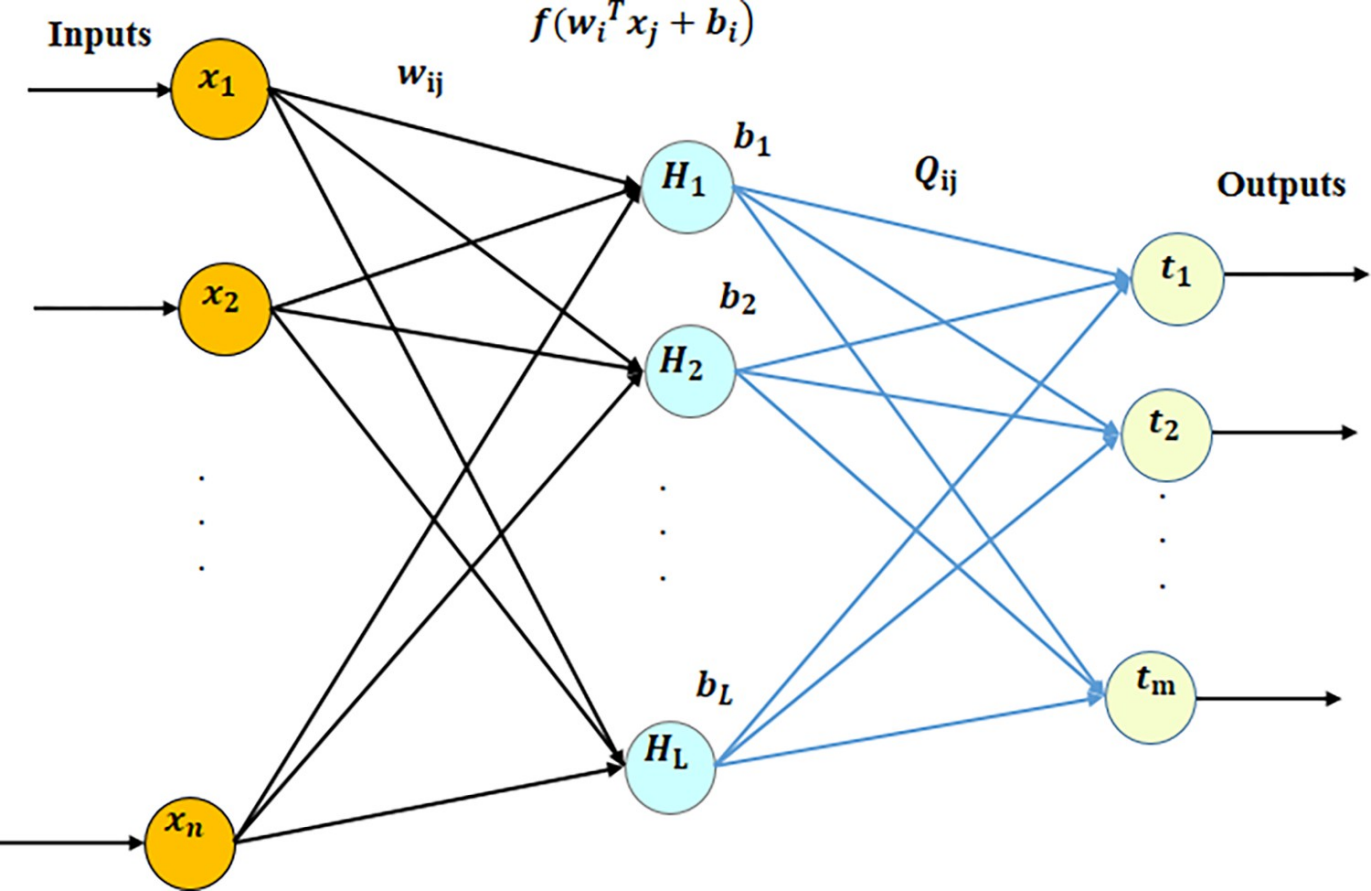
Single-hidden layer neural network [68].

The ELM initially assigns random weights and biases to the input layer and then subsequently computes the output layer weights based on these randomly generated values. The algorithm under consideration exhibits a higher rate of learning and superior performance in comparison to conventional neural network algorithms [17, 21]. In Fig 2, you can see a typical Single-layer Neural Network (SLNN). In this diagram, "n" refers to the number of neurons in the input layer, "L" represents the number of neurons in the hidden layer, and "m" stands for the number of neurons in the output layer.

The formulation of the activation function can be expressed in the following manner [21, 69]:

$$T_{j}=\sum_{i=1}^{l}O_{i}g(w_{i},b_{i},x_{i})$$y (1)

In the given context, the symbol *w*_*i*_ represents the weight of the input connection, *b*_*i*_ denotes the bias of the *i*^*th*^ hidden neuron, *x*_*i*_ means the weight of the output connection, and ***T***_*j*_ represents the final output of the ELM. Eq (**1**) can be written using a matrix format, as demonstrated in the following Eq (**2**) [21, 69]:

$$T^{T}=HO$$ (2)

The transpose of matrix **T** is denoted by ***T***^*T*^. The matrices **H** and O can be represented in the following manner [21, 69]:

$$H=\left[\begin{array}{ccc} g(w_{1},b_{1},x_{1})g(w_{2},b_{2},x_{1}) & \cdots & g(w_{l},b_{l},x_{1})\\ \vdots & \ddots & \vdots \\ g(w_{1},b_{1},x_{\beta })g(w_{2},b_{2},x_{\beta }) & \cdots & g(w_{l},b_{l},x_{\beta }) \end{array}\right]$$ (3)

$$O={\left[O_{1},O_{2},\ldots ,O_{l}\right]}^{T}$$ (4)

The main objective of ELM training is to mitigate training errors. The Classical ELM methodology encompasses the utilization of randomly selected input biases and weights in conjunction with an activation function that possesses infinite differentiability [64]. The output weight (O) is obtained by maximizing the least-squares value in Eq (**5**), and the solution can be derived as shown in Eq (**6**) [21, 69]:

$$\begin{array}{r} \min\\ O \end{array}\left\|HO-T^{T}\right\|$$ (5)

$$\hat{O}=H^{+}T^{T}$$ (6)

The modified Moore-Penrose inverse of the matrix **H** is denoted as **H**^+^.

The performance of ELM is influenced by the quantity of hidden layer neurons and the duration of training epochs. In order to find the most effective number of hidden neurons, an experiment was conducted by varying the number of hidden neurons while keeping the number of training epochs constant at 30. An experiment with the Root Mean Square Error (RMSE) found the best number of hidden neurons. The number of hidden neurons varied while keeping the number of training epochs at 30. to evaluate the performance of the ELM. The final structure of the suggested model consisted of 1048 input neurons, 128 hidden neurons, and six output neurons, which were determined depending on the number of classes.

Nevertheless, the instability of the canonical ELM in real-world engineering issues might be attributed to the random values assigned to input weights and biases. Additionally, it has been suggested that the ELM may necessitate a larger quantity of hidden neurons due to the stochastic determination of the input weights and hidden biases [70, 71]. Hence, optimization methods can be utilized to adjust input weights and biases to stabilize the results. In the subsequent section, it is suggested that GWO be used to change the input weights and biases of ELM.

### 3.4 Gray Wolf Optimization

**Fig 3:**
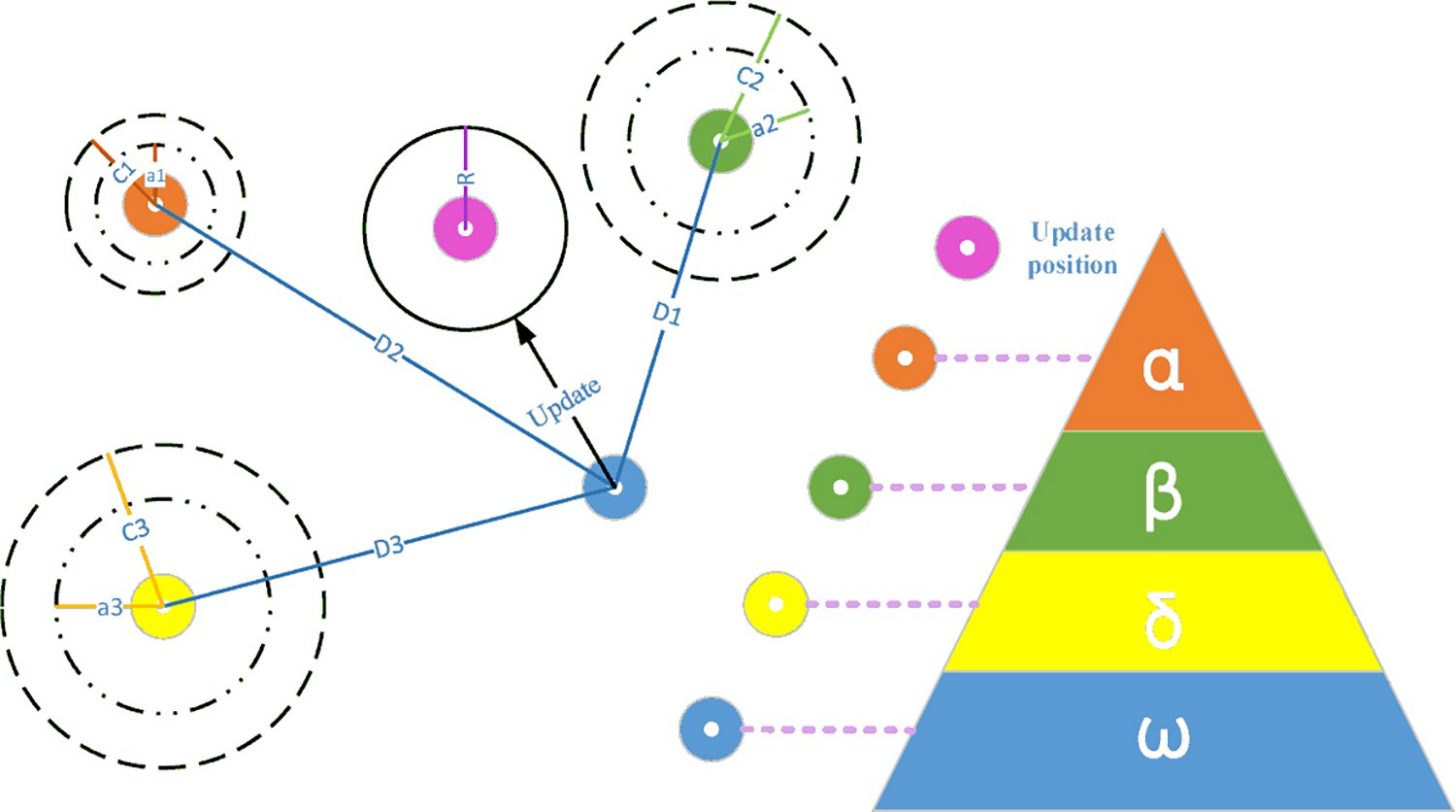
Diagram of the GWO algorithm [72].

GWO draws inspiration from the hierarchical structure and hunting patterns observed in gray wolf communities. The system utilizes mathematical modeling to simulate the various processes of optimizing gray wolf populations, including tracking, surrounding, hunting, and attacking. The hunting procedure of the gray wolf has three distinct stages: social hierarchy stratification, encircling the prey, and attacking the prey [21, 72]. Fig 3 shows a diagram of the GWO algorithm.

#### 3.4.1 Social hierarchy

Gray wolves are highly gregarious animals occupying the apex of the food chain, adhering to a rigid social dominance structure. The optimal solution is denoted as α, whereas the subsequent keys of lesser quality are denoted as β for the second-best, δ for the third-best, and ω for the remaining solutions [21, 72].

#### 3.4.2 Encircling the prey

Gray wolves’ encircling behavior during hunting involves forming a circular arrangement around their prey. To provide a mathematical representation of this behavior, the equations that follow are employed [21, 72]:

$$X(t)=X_{p}(t)-A.|C.X_{p}(t)-X(t)|$$ (7)

$$A=2\alpha r_{1}-\alpha$$ (8)

$$C=2.r_{2}$$ (9)

$$\alpha =2-2\frac{t}{Max\_iter}$$ (10)

Let *X* represent the location vector of the gray wolf, *Xp* represent the position vectors of prey, *t* represent the current iteration, *A* and *C* represent coefficient vectors, *r*_1_ and *r*_2_ represent random vectors in the interval [0, 1]^n^ raised to the power of n, *a* represents the distance control parameter, which linearly decreases from 2 to 0 across the duration of iterations, and Max_iter represents the maximum number of iterations [21, 72].

#### 3.4.3 Attacking the prey

Gray wolves can discern the whereabouts of potential prey, with the search procedure facilitated mainly by the leadership of alpha, beta, and delta wolves. During each iteration, the three wolves with the highest fitness values (represented as *α, β*, and *δ*) are preserved in the current population. Subsequently, the positions of the remaining search agents are updated based on their respective position information. The subsequent equations are suggested about this matter [21, 72]:

$$X_{1}=X_{\alpha }-A_{1}.|C_{1}.X_{\alpha }-X|$$ (11)

$$X_{2}=X_{\beta }-A_{2}.|C_{2}.X_{\beta }-X|$$ (12)

$$X_{3}=X_{\delta }-A_{1}.|C_{3}.X_{\delta }-X|$$ (13)

$$X(t+1)=\frac{X_{1}(t)+X_{2}(t)+X_{3}(t)}{3}$$ (14)

**Fig 4:**
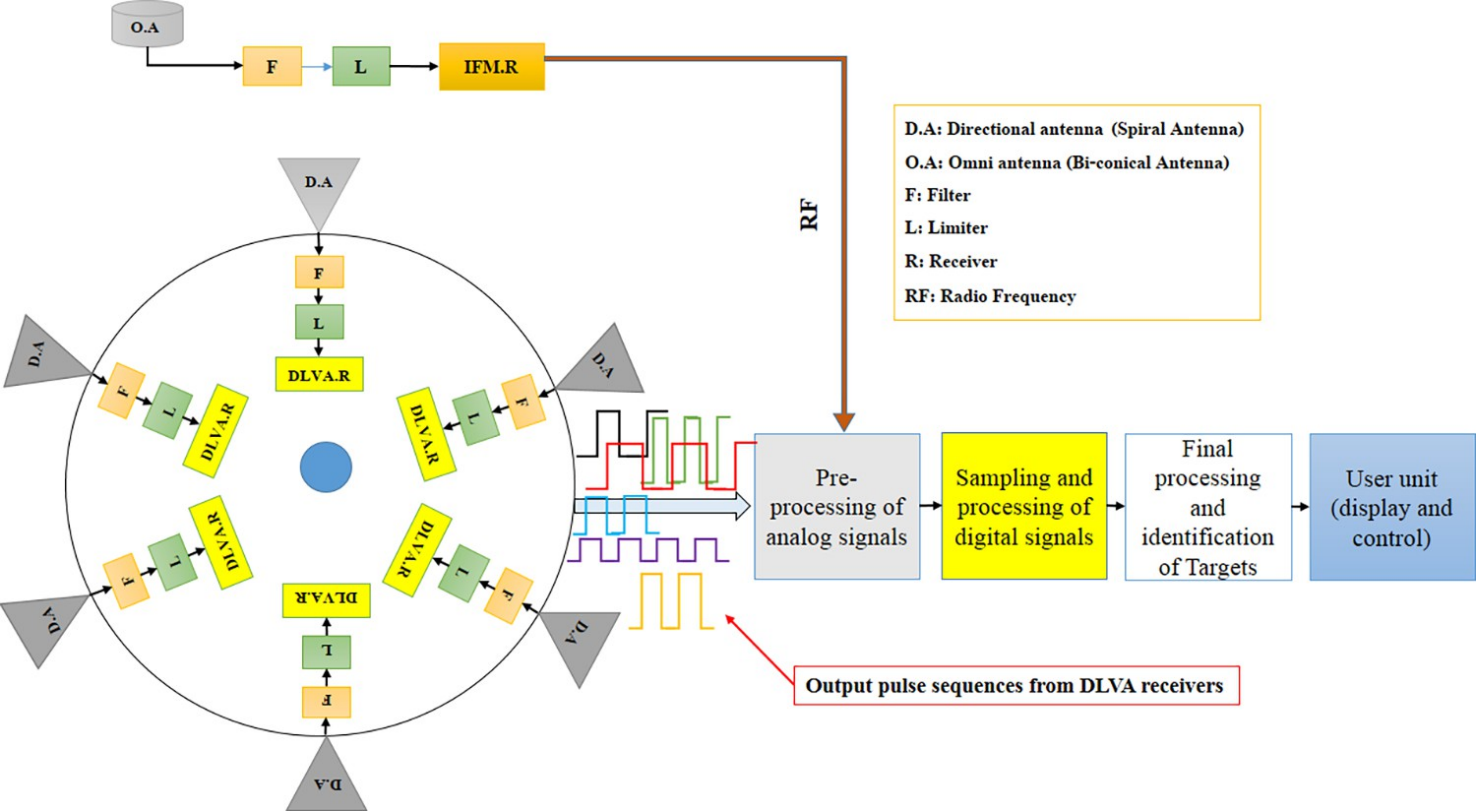
Overview of the designed system’s operational process.

In the given equation, *X*_*α*_, *X*_*β*_, and *X*_*δ*_ represent the position vectors of α, β, and δ wolves, respectively. The computations for *A*_1_, *A*_2_, and *A*_3_ are analogous to those for *A*, while the counts for *C*_1_, *C*_2_, and *C*_3_ are analogous to those for *C*. The equations *D*_*α*_ = *C*_1_*X*_*α*_−*X*, *D*_*β*_ = *C*_2_*X*_*β*_−*X*, and *D*_*δ*_ = *C*_3_*X*_*δ*_−*X* are used to denote the distance between the current candidate wolves and the top three wolves. As depicted in Fig 4, the candidate solution ultimately resides within the random circle delineated by *α, β*, and *δ*. Subsequently, under the supervision of the three most proficient wolves, the remaining contenders randomly adjust their locations in proximity to the prey. The individuals commence their hunt for information regarding the position of their prey in a disorganized manner, focusing their efforts on launching an attack on the prey.

## 4. The suggested procedure

The suggested procedure, called DCNN_ELM_GWO, incorporates a hybrid model consisting of a DCNN combined with ELM and GWO techniques.

The methodology proposed, called DCNN_ELM_GWO, and integrates a hybrid DCNN model with ELM and GWO methods. This research presents a novel three-step process for identifying six prevalent PRIM forms. DL approaches are limited in addressing these aspects due to the substantial time required for training and fine-tuning the model parameters. This methodology involves a three-step process. Firstly, a DCNN is trained as a feature extractor. Secondly, an ELM is employed for real-time pattern identification. The primary approach ELM uses involves randomly adjusting input weights and biases. However, this practice needs to improve the network’s stability and dependability, as the network’s performance relies heavily on the initial adjustment of weights and biases. Consequently, this study proposes utilizing the GWO algorithm to enhance outcomes and bolster network reliability, all while preserving real-time capabilities.

Transfer learning has been employed to train the targeted neural networks. This approach involves using pre-trained weights from DCNNs trained on the ImageNet dataset, which includes a wide variety of classes. Only the fully connected layers at the network’s end are trained, while the remaining layers retain pre-trained weights. The fully connected layers that have been substituted in all networks exhibit uniformity, as they consist of a fully connected layer comprising 1024 neurons with a Relu activation function, a fully connected layer comprising 128 neurons with a Relu activation function, and a fully connected layer comprising six neurons, which aligns with the output classes, utilizing a softmax activation function.

### 4.1. Investigation of the empirical dataset

This study has generated a unique dataset of PRI radar signals to evaluate the suggested methodology, marking the first instance of such an endeavor. The study was carried out at Imam Khomeini Marine University, located in Nowshahr, throughout the period spanning from September to December 2020. To fulfill the intended objective, the system necessary for this task was meticulously devised and deployed within an area characterized by a substantial concentration of radar signals, where it remained operational for eight months. To achieve the desired functions and fulfill the specified criteria, electronic support systems are typically structured into several key components: radio antennas and receivers, hardware, control, and power supply units, processing units including processors, software, and processor units, as well as user consoles.

The passive approach receives, detects, processes, and analyzes radar signals within the 2–18 GHz frequency range. Based on the specified objectives and needs, the system comprises two primary components: the external component, which encompasses antennas and radio receivers, and the inside, part, which includes processor sets and hardware units. The establishment of connectivity between these two components is facilitated through cable interfaces. The antenna arrangement pattern design involves considering each antenna’s radiation pattern and coverage and determining the appropriate number of antennas needed to form an array that covers 360 degrees. The system processes the output signals from the receivers in real time, depending on the particular type of receiver.

Fig 4 illustrates the overall operational process of the system that has been designed. This figure provides a visual representation of the comprehensive operational process of the developed system, depicting the sequence of operations and interactions within the system.

**Fig 5:**
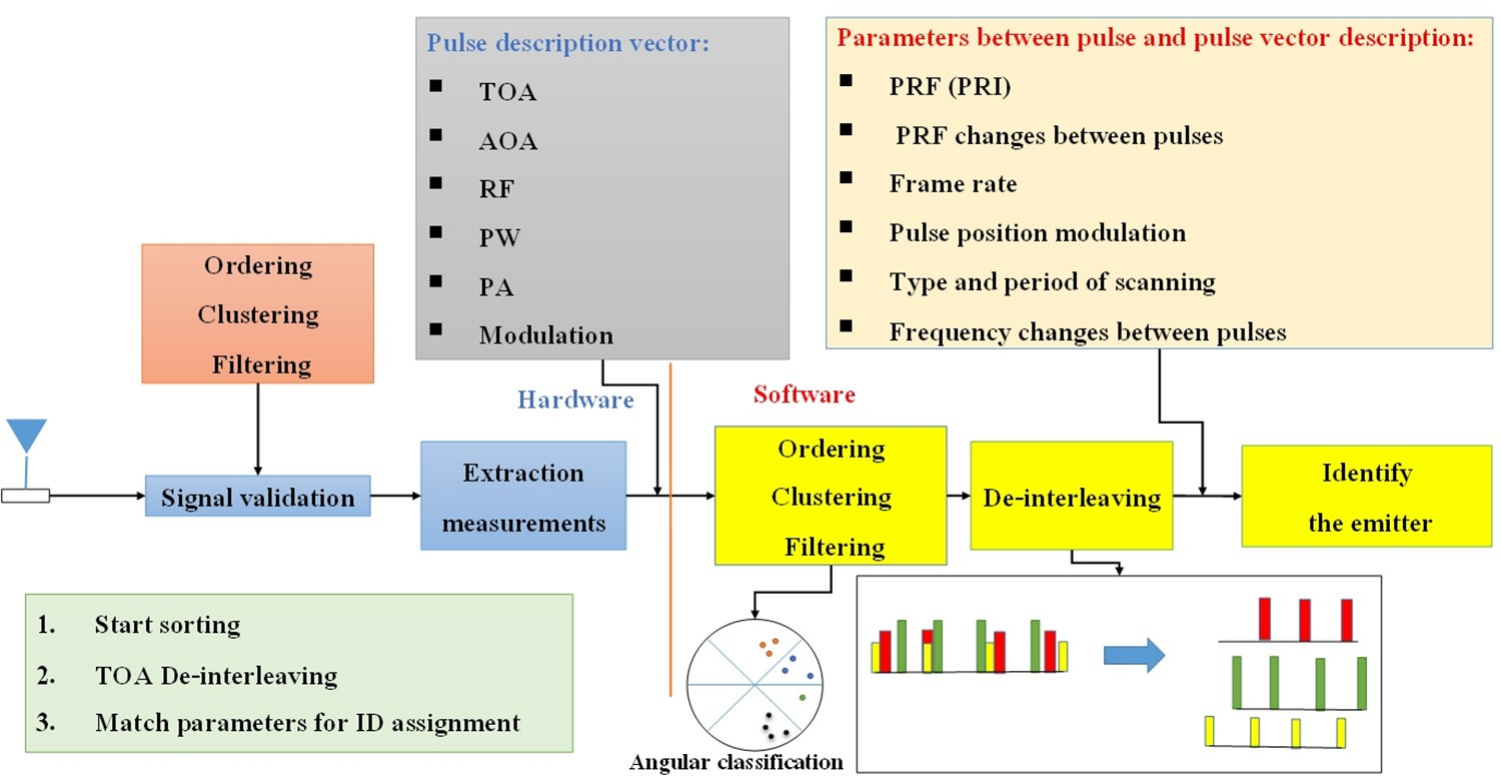
Comprehensive block diagram of system processing activities.

Fig 5 illustrates the overarching block structure that represents the several processing processes conducted by the system. During the hardware and software processing stages, the system monitor presents the parameters of the extracted targets inside the domain of processors.

In the software processing component of the system, the first phase entails doing activities such as classifying, filtering, and splitting the information. Consequently, an investigation into the segregation of pulses is conducted. After the successful completion of this stage, the subsequent supplementary parameters are obtained. Identifying the target involves measuring many properties.

ELINT or ESM systems receive radar signals and subsequently analyze the characteristics of each detected pulse.

This work introduces the utilization of PRI sound for identifying its modulation type. To achieve this objective, the PRI sequence obtained from a width hold signal (WHS) module is subjected to compression, ensuring that the amplitude of the sequence remains consistent. Subsequently, the compressed line is inputted into the sound card, and the resulting sound produced by the series is recorded through the speaker output.

The initial audio data exhibits a significant amount of noise. The technique employed in the study referenced as [11] has been utilized to mitigate unwanted disturbances in the initial audio dataset.

When presented with a waveform including both a signal and background noise (Sn), as well as a sample audio clip derived from the same or a comparable waveform but consisting solely of background noise (N), The algorithm is outlined as follows [73]:

1. Calculate the short-time Fourier transform for a given N (spec_n_) value.
2. To calculate the statistical measures for each frequency component across time, we must determine the mean and standard deviation of spec_n_.
3. To calculate the short-time Fourier transform of Sn (spec_n_), perform the necessary computations.
4. The mean and standard deviation of the spec_n_ should be utilized to determine the threshold noise level for each frequency component.
5. To create a mask over specifications, it is necessary to consider the strength of the specifications and the predetermined thresholds from the spec_n_ dataset.
6. The mask should be applied evenly throughout both frequency and time domains.
7. The mask should be applied to the specifications to eliminate any noise present.
8. The inverse short-time Fourier transform is computed across the given specifications to obtain a de-noised time-domain signal.

**Fig 6:**
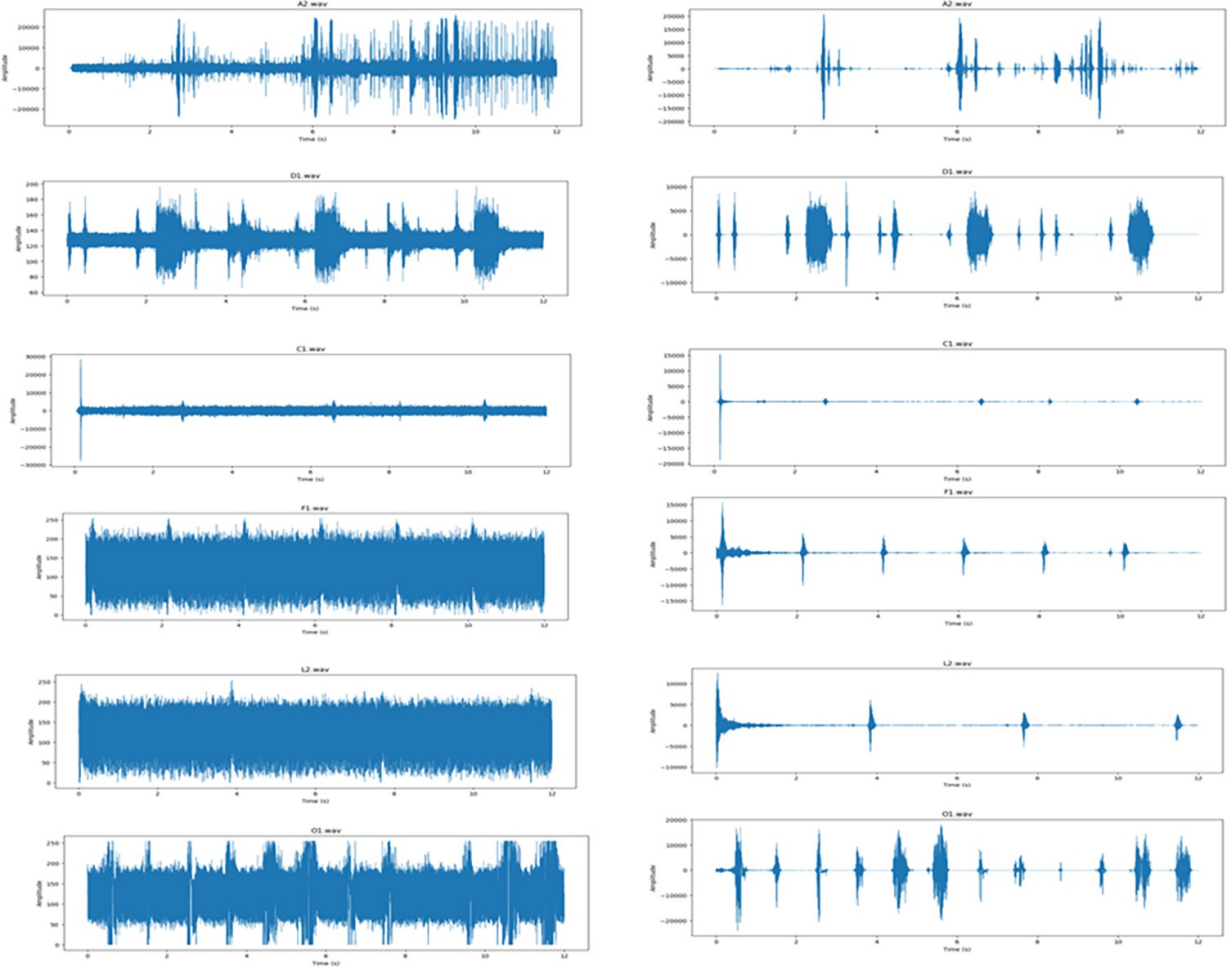
Comparison of Sound Data, (a) the original sound data with inherent noise, and (b) the sound data post the noise removal process.

Fig 6 illustrates the initial sound data and the sound data after the application of a de-noising technique for noise removal.

**Fig 7:**
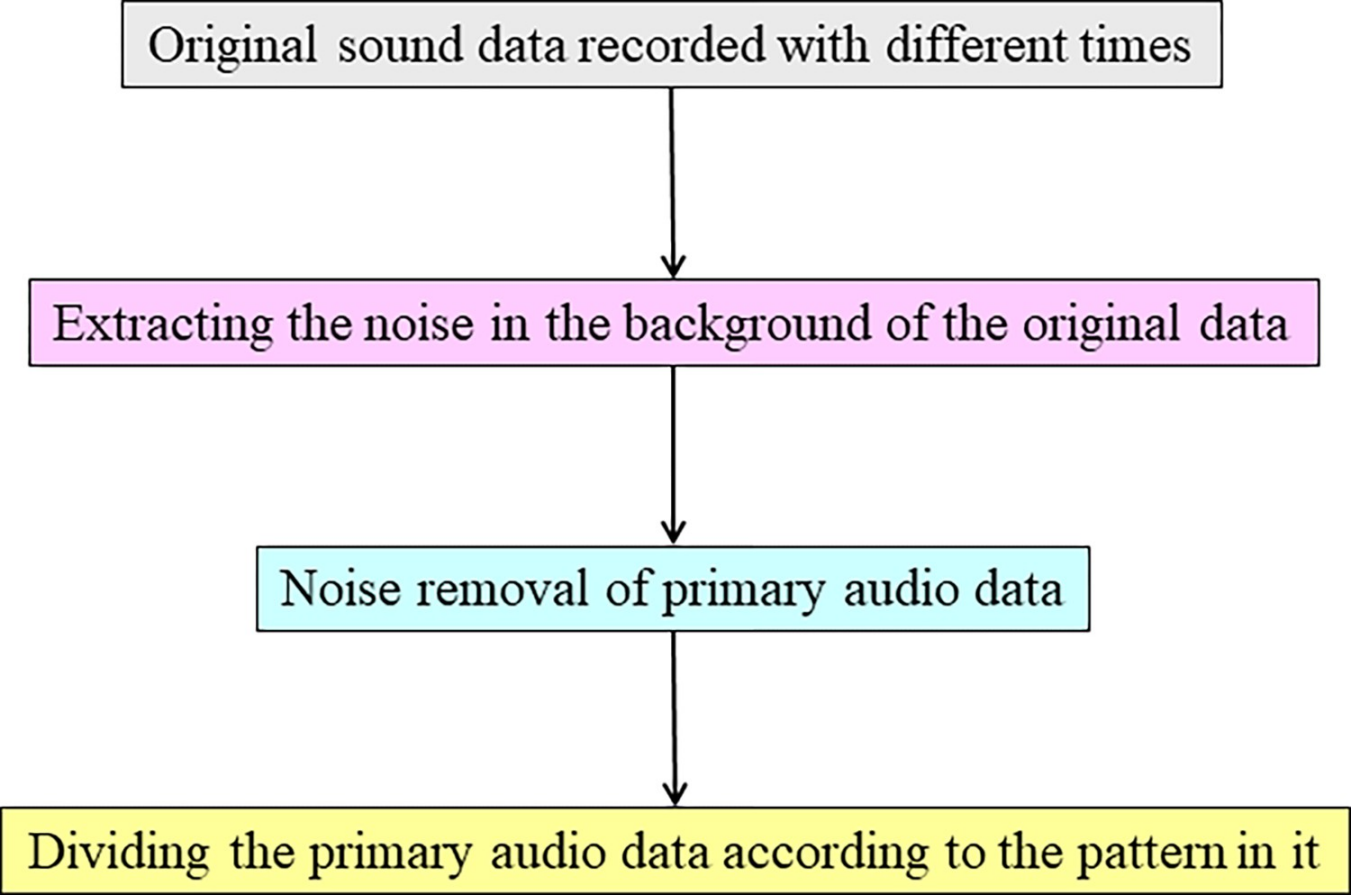
Block diagram of dataset preparation.

The initial acoustic data exhibited temporal variations. A thorough analysis was conducted on the noise-free data after eliminating the extraneous elements from the original dataset. This analysis involved organizing the data into distinct segments of varying lengths based on the repeat duration of patterns seen within each audio data class. The subject matter is partitioned into four parts and allocated to their respective categories. The several selected components exhibit no overlap with one another. One hundred eight audio data samples, representing six distinct classes, were extracted for the collection. Fig 7 presents the block diagram illustrating the preparation of the current dataset. This figure provides a visual representation of the process undertaken for the preparation of the current dataset, illustrating the sequential steps and components involved in organizing and refining the data.

**Fig 8:**
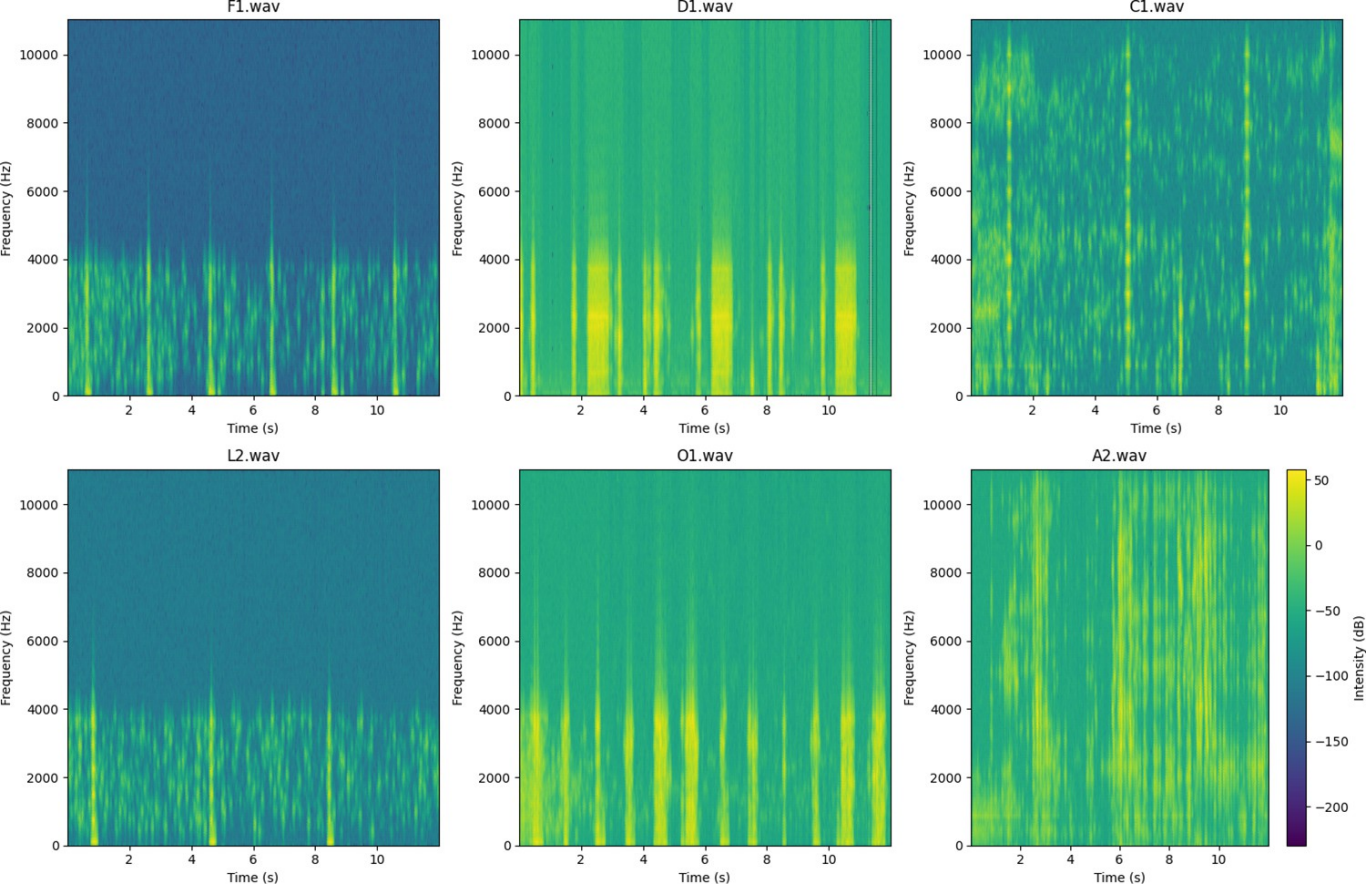
Spectrogram images of sample data in each class.

The spectrogram images of the preexisting audio data have been extracted to facilitate the anticipated networks’ training process. Subsequently, these photos are employed to train the neural networks. Fig 8 presents spectrogram images representing the variability and characteristics of sample data within each designated class, allowing visual interpretation of the dataset’s frequency and time domain features.

The dataset consists of a total of 108 data points, encompassing six distinct types of modulation. The data points were split into three subsets: 70% for training, 15% for validation, and 15% for testing. Data augmentation techniques have been employed to augment the existing dataset. Indeed, this strategy solely resulted in a quadrupling of the training data. Data augmentation has used two methods for introducing noise and jittering in the temporal domain. Table 3 provides comprehensive information about the dataset used in this study, including specifications and characteristics relevant to the research.

**Table 3:** Details of the employed dataset.

PRI Type	Number
Periodic	**10**
Stagger	**17**
Sliding	**17**
Dwell & switch	**21**
Constant	**22**
Jitter	**21**

### 4.2. Evaluation measurements

To assess the classification performance of our technique, we utilized six commonly utilized metrics: sensitivity, specificity, accuracy, precision, Matthew’s correlation coefficient (MCC), and F1 score [74]. The evaluation of the performance of a classification job often involves the use of accuracy and F1 score, which are commonly employed metrics generated from confusion matrices. Our study used these metrics to compare our results with other benchmark models [75]. Nevertheless, it is essential to note that these statistical techniques have the potential to unveil too optimistic results, mainly when used in datasets that need to be balanced appropriately [76]. The Matthews Correlation Coefficient (MCC), in contrast, is a reliable statistical measure that assigns a high score only when the prediction demonstrates strong performance across all four categories of the confusion matrix (true positives, false negatives, false positives, and true negatives), relative to the proportions of positive and negative instances in the dataset. The primary objective of specificity is often to demonstrate or evaluate the test’s ability to exclude the presence of a specific illness with accuracy effectively. Assessing a test’s classification accuracy is of utmost importance when the cost of a false positive could be prohibitively high. The statistic above holds significant importance in the context of military applications. An increase in sensitivity is often associated with a decrease in specificity, and vice versa, creating an inverse relationship between the two. As stated by the source referenced [74], sensitivity and specificity are considered more reliable metrics than accuracy when evaluating the effectiveness of a test. The calculations can be determined by employing the subsequent formulas [77]:

$$Specificity=\frac{TN}{TN+FP}$$ (15)

$$Senseitivity=\frac{TP}{TP+FN}$$ (16)

$$Accuracy=\frac{TP+TN}{TP+FP+FN+TN}$$ (17)

$$F_{1}-score=\frac{TP}{TP+\frac{1}{2}(FP+FN)}$$ (18)

$$Precision=\frac{TP}{TP+FP}$$ (19)

$$MCC=\frac{TP\times TN-TP\times FN}{\sqrt{\left(FP+TP)\times (FP+TN)\times (FN+TP)\times (FN+TN\right)}}$$ (20)

TN refers to the count of instances that are true negatives. Whereas TP represents the number of actual positive cases. FP denotes the number of false-positive cases, while FN denotes the number of false-negative cases.

The efficacy of the suggested methodology is assessed through three distinct investigations, which are outlined as follows:

1. Firstly, the performance of eight deep convolutional neural networks (DCNN) types is assessed and compared using the dataset.
2. Additionally, an investigation is conducted on the performance of integrating DCNNs with ELM using the dataset.
3. Additionally, the DCNN-ELMs that have achieved the most favorable outcomes are subjected to optimization using the Grey Wolf Optimization (GWO) algorithm, and their performance is evaluated on the dataset.

The network with the highest levels of accuracy and speed has been unveiled.

The suggested DCNNs were trained using Google Colab’s shared hardware and the T4 graphics card. The DCNN_ELM and DCNN_ELM_GWO networks were trained on shared Google Kolb hardware and CPU due to the absence of shared RAM. The models required are created using Python programming language with Tensorflow and Keras libraries. The total number of Epochs for all networks is set at 30, while the batch size for all networks is standardized to 16. An initial training rate of 0.001 is initially chosen in training neural networks. Subsequently, if the evaluation data’s accuracy does not decline throughout five epochs, the training rate is halved. The minimum value for the training rate is set at 0.00001. The quantification of weights for transfer learning in pre-trained networks is performed using the methodology given by the TensorFlow library.

**Fig 9:**
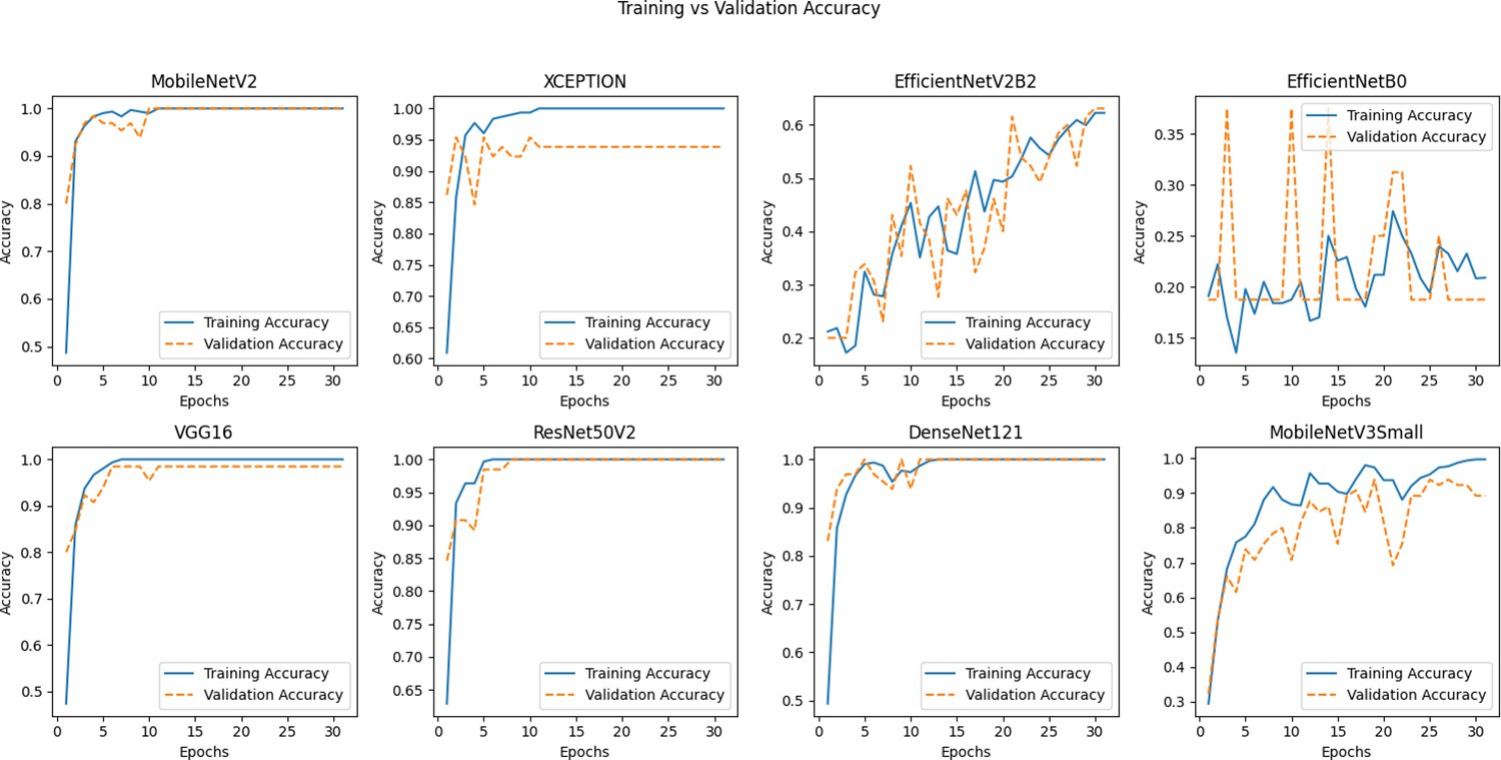
The training diagram of the DCNN approaches.

Fig 9 Training Diagram for DCNN Approaches. This diagram illustrates the various steps involved in the Deep Convolutional Neural Networks (DCNNs) training process, providing insights into the implemented methodologies.

**Fig 10:**
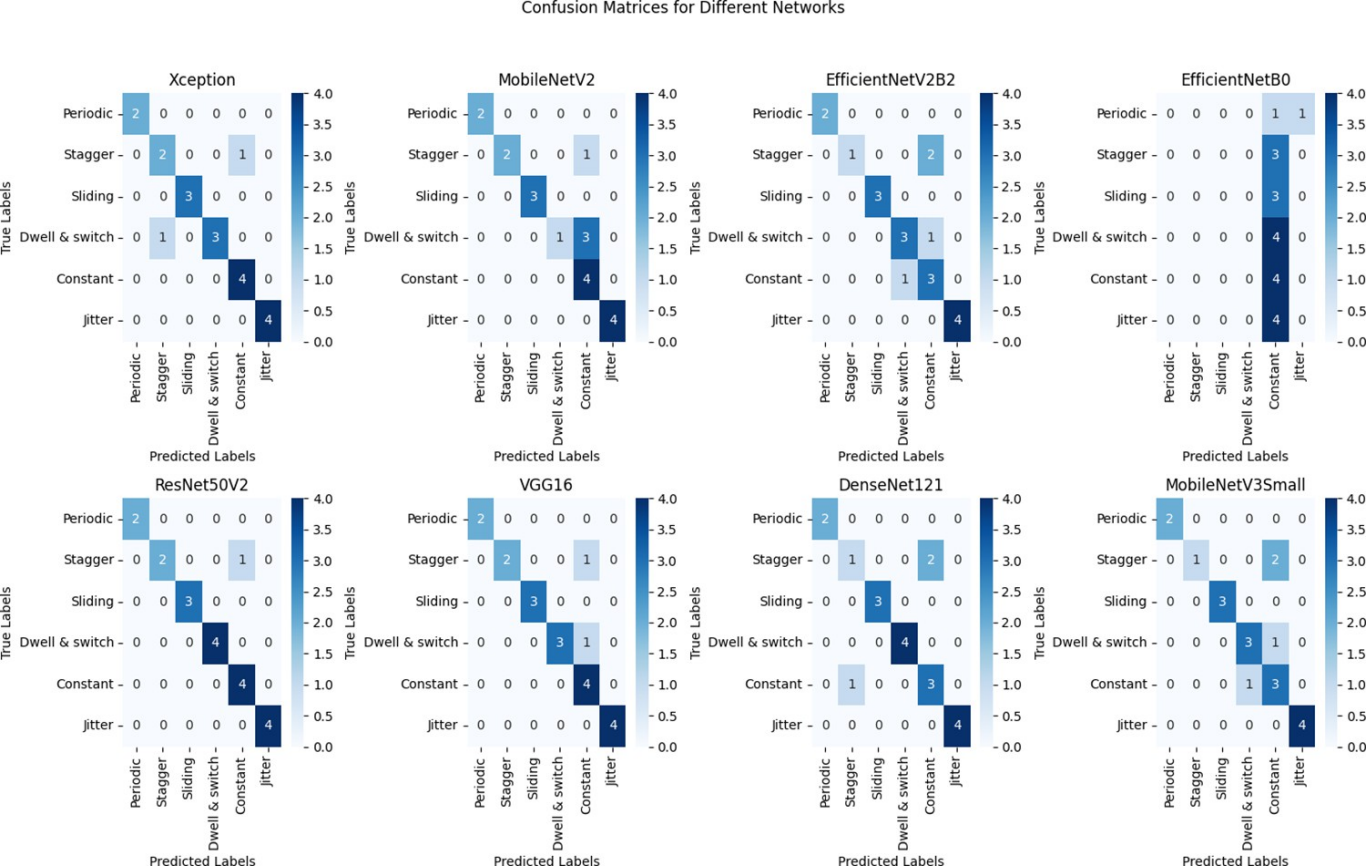
The outcomes of the confusion matrix for the DCNNs techniques.

Fig 10 confusion matrix findings for each neural network. This figure presents the confusion matrix results for each implemented neural network, illustrating the classification performance and accuracy of the models.

**Fig 11:**
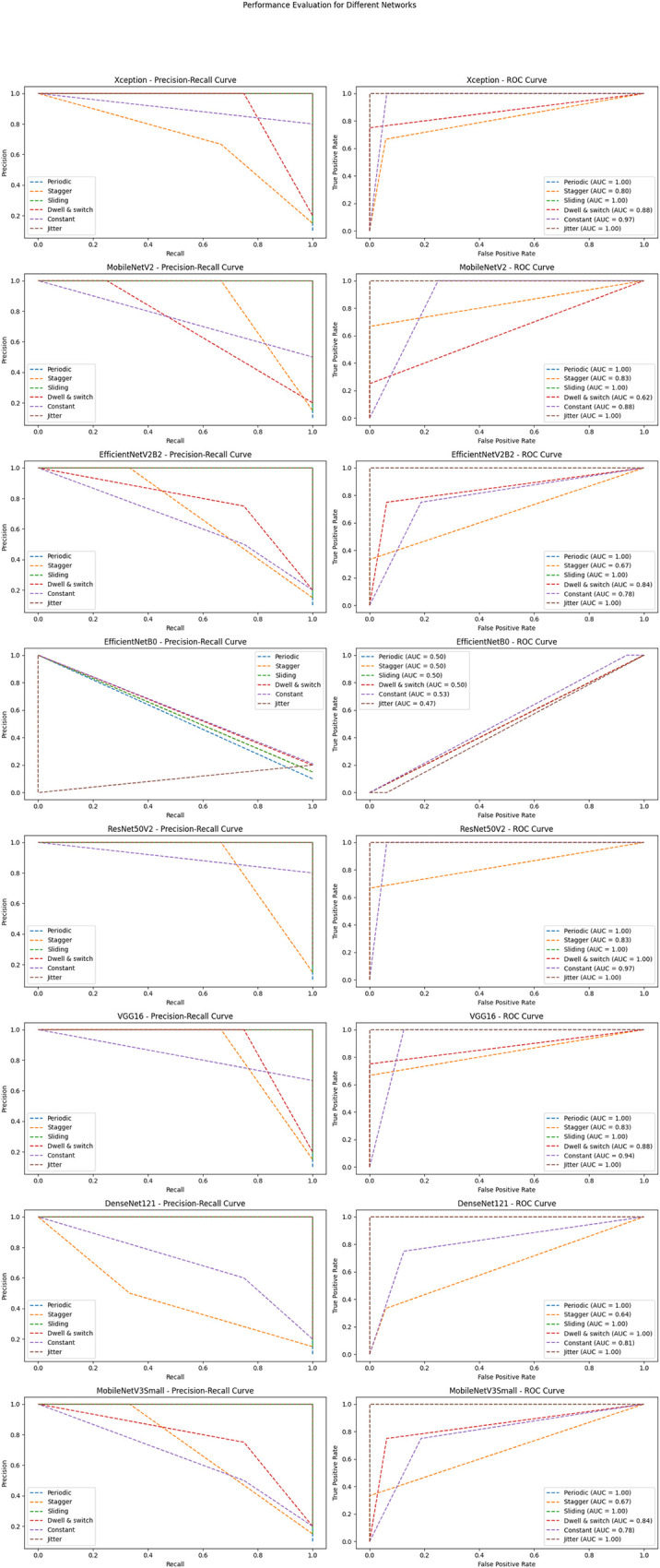
The precision-recall and ROC curves for approaches employing DCNNs.

Fig 11 illustrates the precision-recall and receiver operating characteristic (ROC) curves for approaches employing DCNNs.

Table 4 compares the classification results of various DCNN approaches, emphasizing performance and accuracy differences.

**Table 4:** Comparative analysis of DCNNs classification outcomes.

R	Model	Sensitivity (%)	Specificity (%)	Precision (%)	F1 Score (%)	Accuracy (%)	MCC (%)
1	MobileNetV2	81.94	95.83	91.66	81.11	80	81.07
2	Xception	90.27	97.97	91.11	90.21	90	88.56
3	EfficientNetB0	16.66	83.33	3.50	5.79	20	15
4	EfficientNetV2B2	80.55	95.83	87.5	80.83	80	78.74
5	VGG16	90.27	97.91	94.44	90.95	90	89.95
6	ResNet50V2	94.44	98.95	96.66	94.81	95	94.32
7	MobileNetV3Small	80.55	95.83	87.5	80.83	80	78.74
8	DenseNet121	84.72	96.93	85	84.44	85	81.73

Table 5 outlines the complexity analysis of the DCNN methods, detailing the computational cost and resources required by each approach.

**Table 5:** Complexity analysis of DCNNs methods.

R	Model	Training time (s)	Each Step per Epoch (ms)	Number of network parameters (million)	FLOPS (million)	Average Rank
1	MobileNetV2	12.13	20	64.3	129	85.26
2	Xception	21.10	32	102.8	206	91.35
3	EfficientNetB0	12.44	20	64.36	129	24.05
4	EfficientNetV2B2	12.03	23	70.7	142	83.90
5	VGG16	10.61	12	25.8	51	92.25
6	ResNet50V2	21.32	31	102.9	206	95.69
7	MobileNetV3Smalle	14.44	24	48.3	96	83.90
8	DenseNet121	10.64	17	51.5	103	86.30

**Fig 12:**
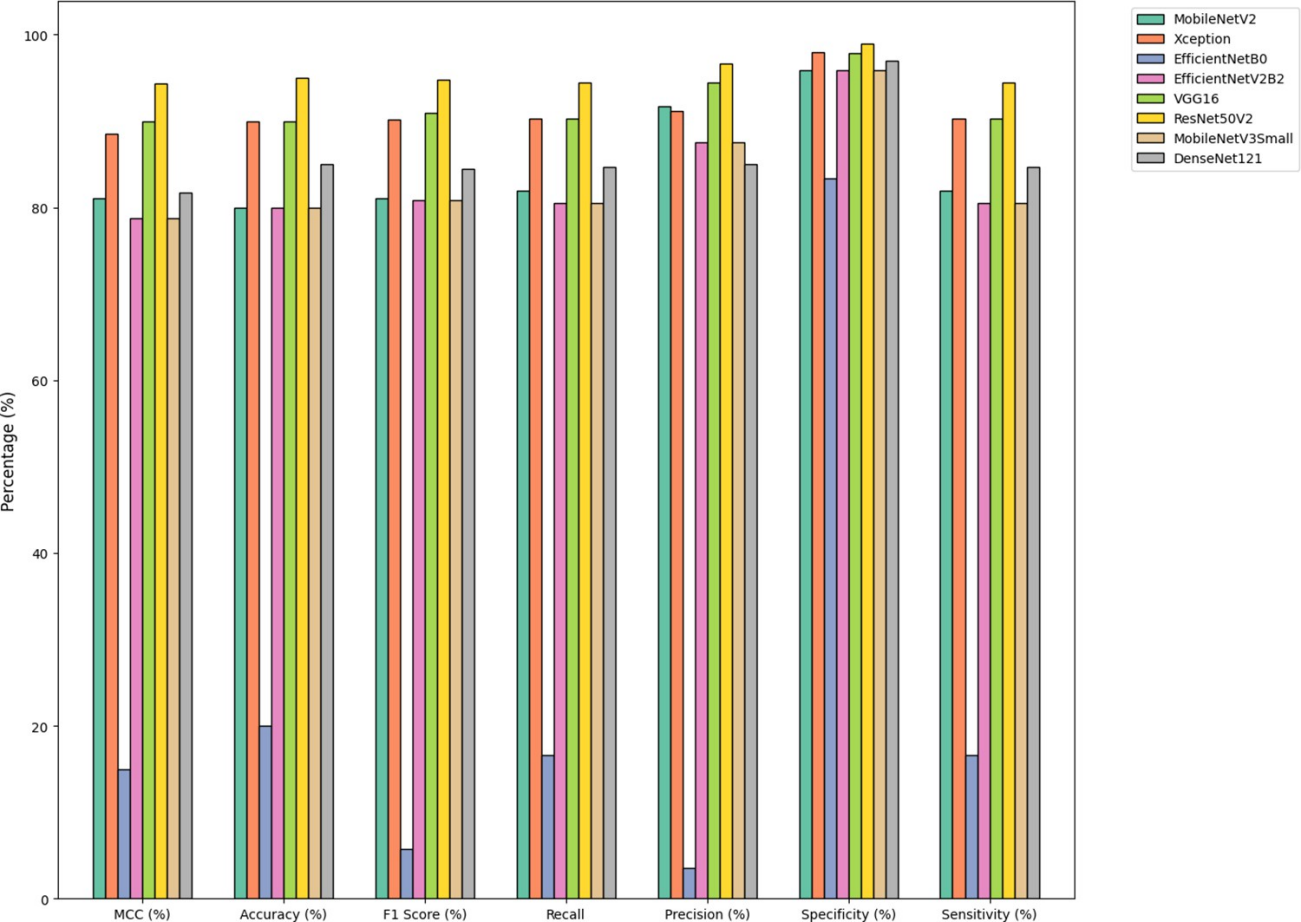
Comparison of computational outcomes of proposed DCNNs.

Fig 12 provides a comparative visualization of the computational outcomes, showcasing the efficiency and effectiveness of the proposed DCNNs in the study.

**Fig 13:**
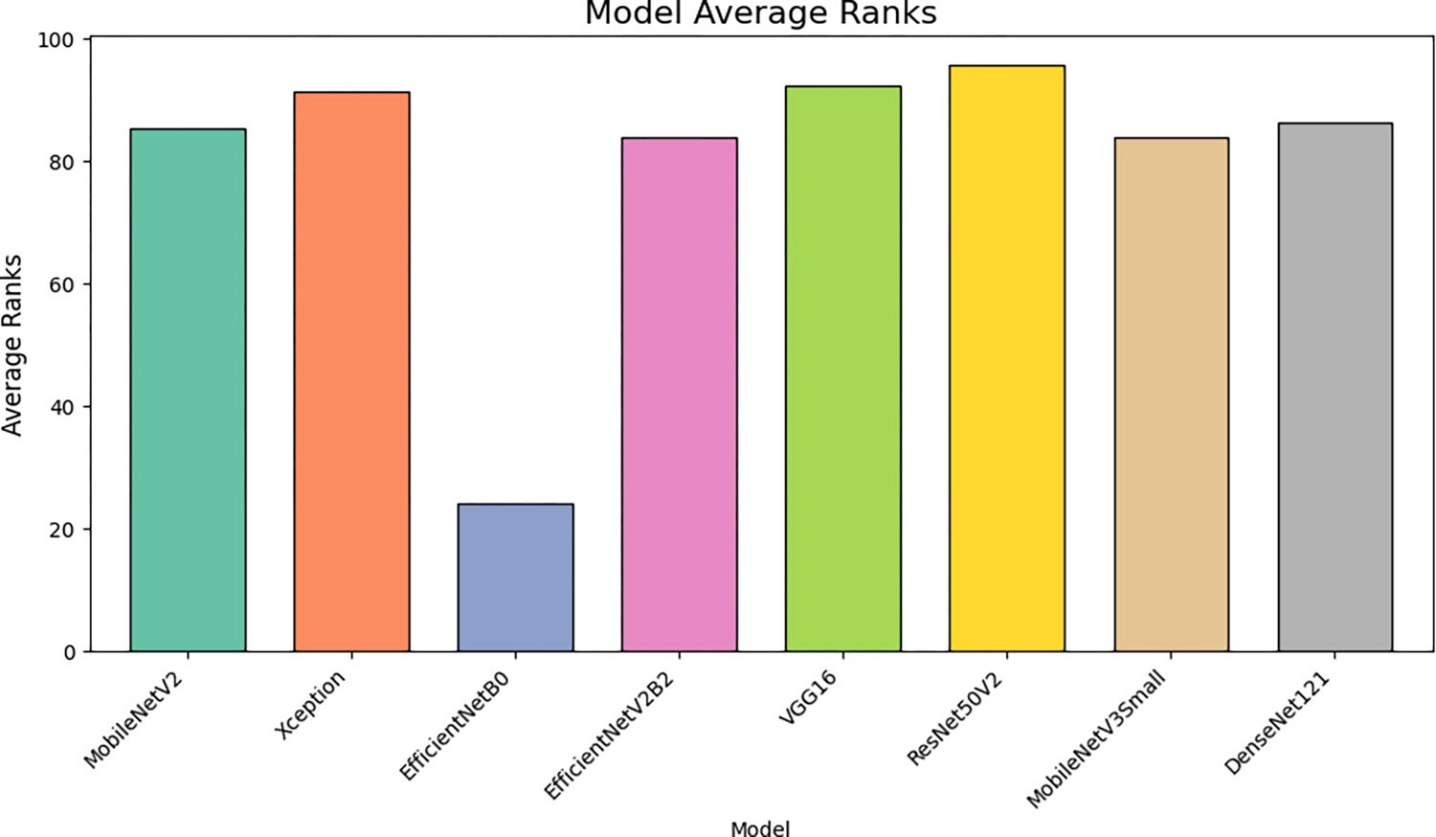
Comparison of average measurement criteria of the suggested method.

Fig 13 Illustrates the comparative analysis of the suggested method’s average measurement criteria, focusing specifically on the average rank, to provide insights into its performance and reliability.

**Fig 14:**
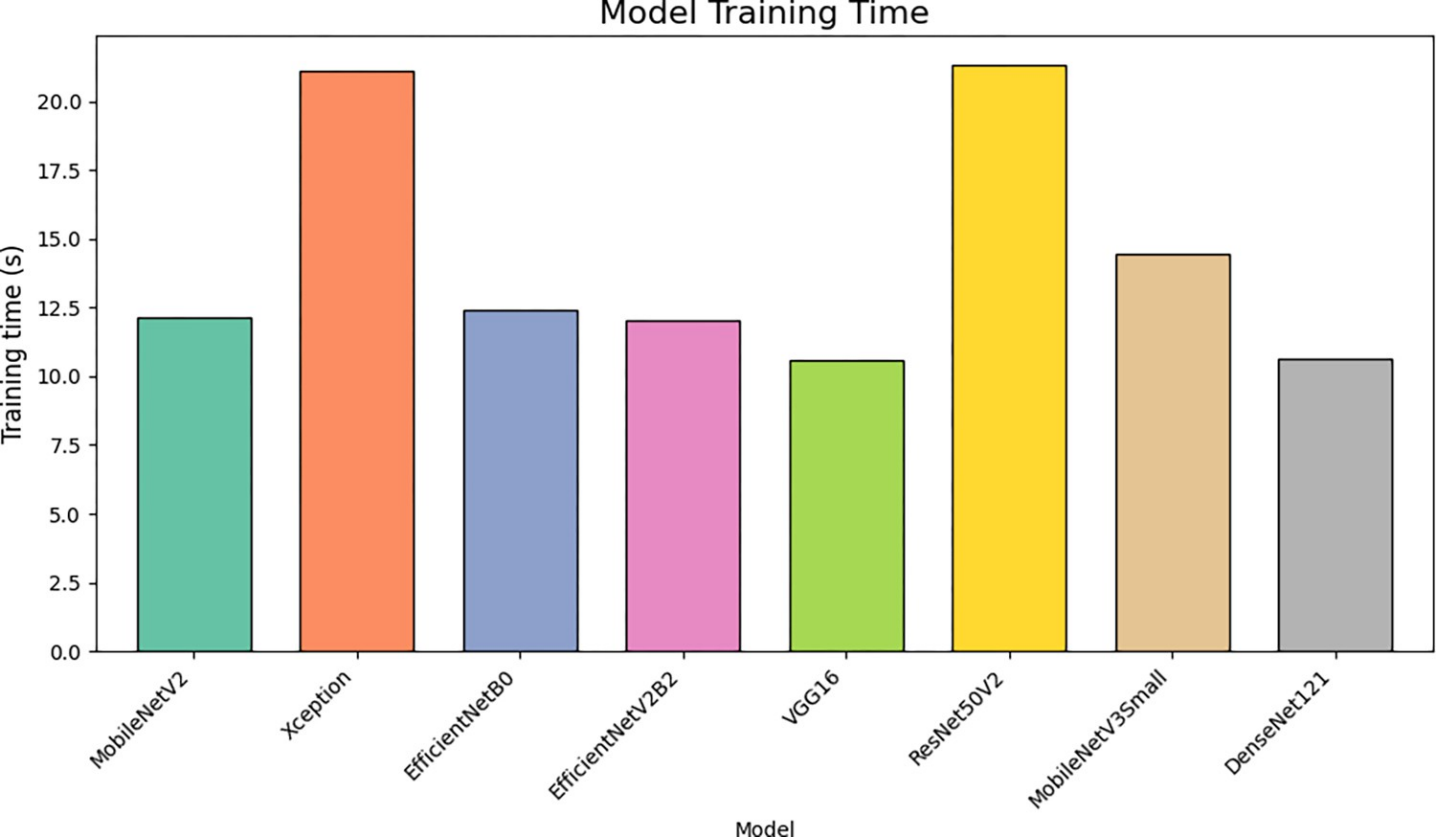
Training time of the proposed DCNNs.

Fig 14 Visualizes the time required to train the proposed DCNNs, providing insights into their computational efficiency.

From the insights garnered from Tables 4 and 5 and Figs 10–14, it is discernible that ResNet50V2 stands out as the optimum performing model, registering the highest scores in all specified metrics, accentuating its aptitude in classification tasks. Conversely, EfficientNetB0 significantly trails in every assessed metric, suggesting it may be relatively inefficient in managing classification tasks when juxtaposed with its peers. Both VGG16 and Xception exhibit exceptional and well-balanced performance, portraying them as reliable across many classification scenarios. Interestingly, VGG16 and DenseNet121 feature lower FLOPS, fewer network parameters, and reduced training times, suggesting they are more economically feasible regarding computational demands and enable faster inference. However, despite being high achievers, ResNet50V2 and Xception incur higher computational overheads due to increased FLOPS and network parameters, potentially necessitating substantial resources and elongating inference times. It is noteworthy that EfficientNetB0, despite its suboptimal performance, presents competitive complexity metrics comparable to MobileNetV2, underscoring the importance of a balanced approach between efficiency and performance. The data brings to light a prominent trade-off between performance and complexity. Models like ResNet50V2, albeit high-performing, are associated with higher computational demands, possibly constraining their applicability in environments with limited resources. Conversely, models such as VGG16 strike a balance, delivering notable performance and lower computational requisites, rendering them adaptable to broader applications.

**Fig 15:**
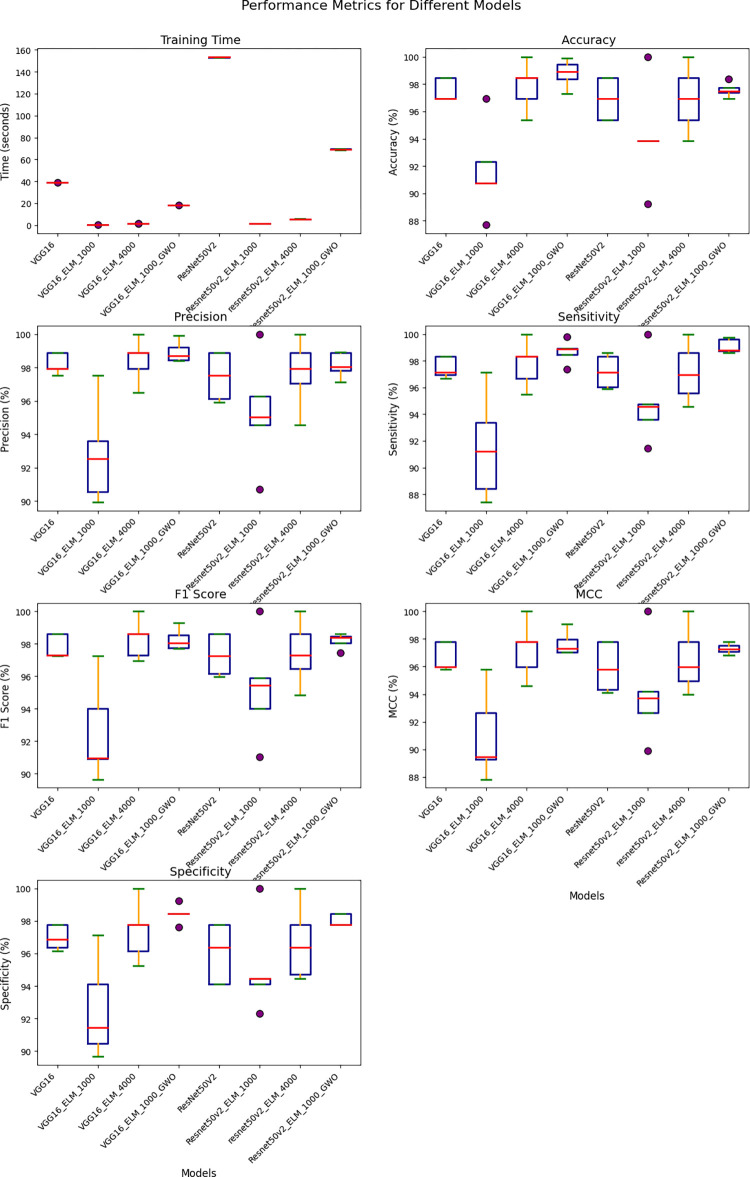
Comparative performance analysis of hybrid DL models using VGG16 and ResNet50V2 with ELM and GWO integration.

**Fig 16:**
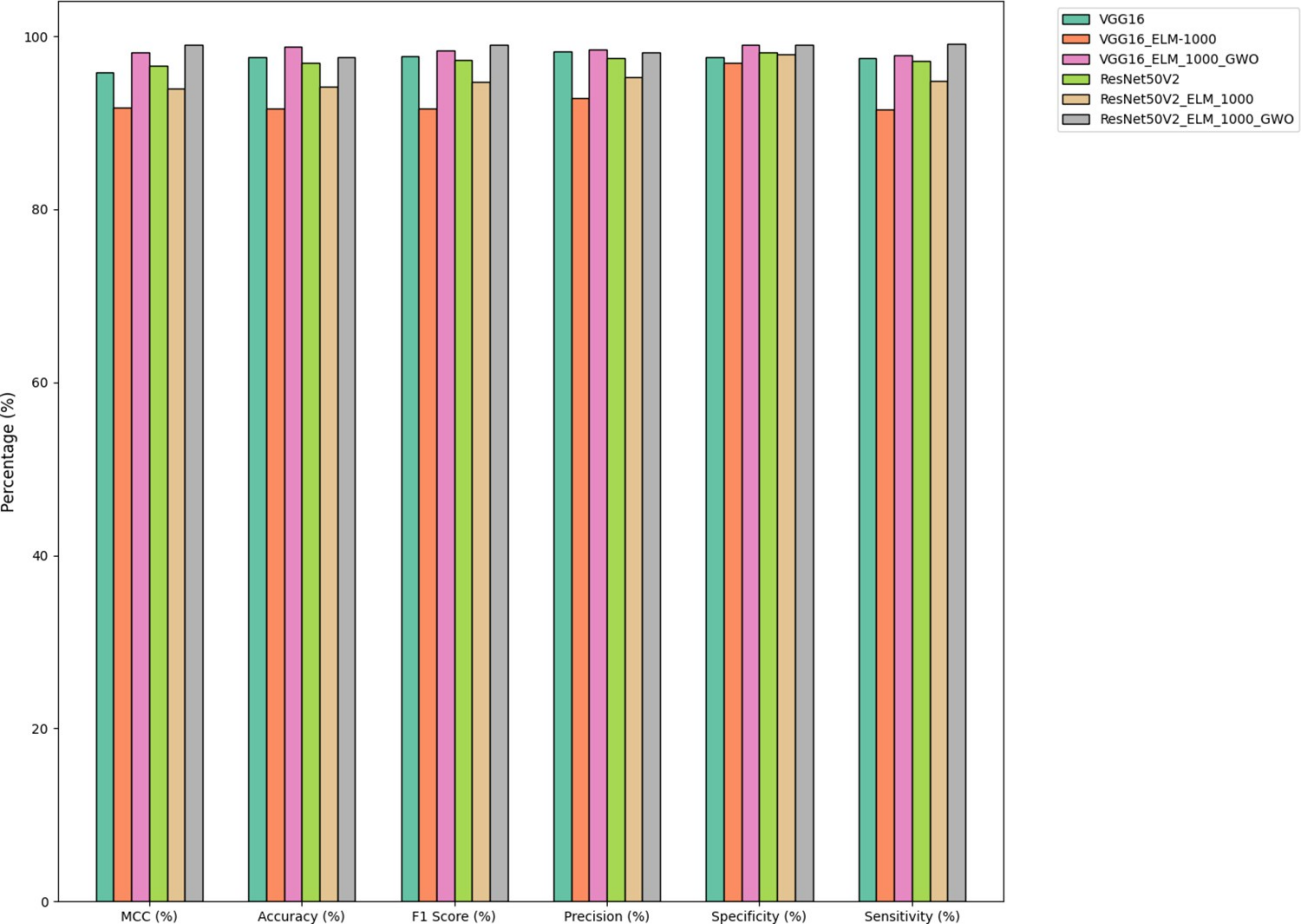
Comparative performance of various approaches.

**Fig 17:**
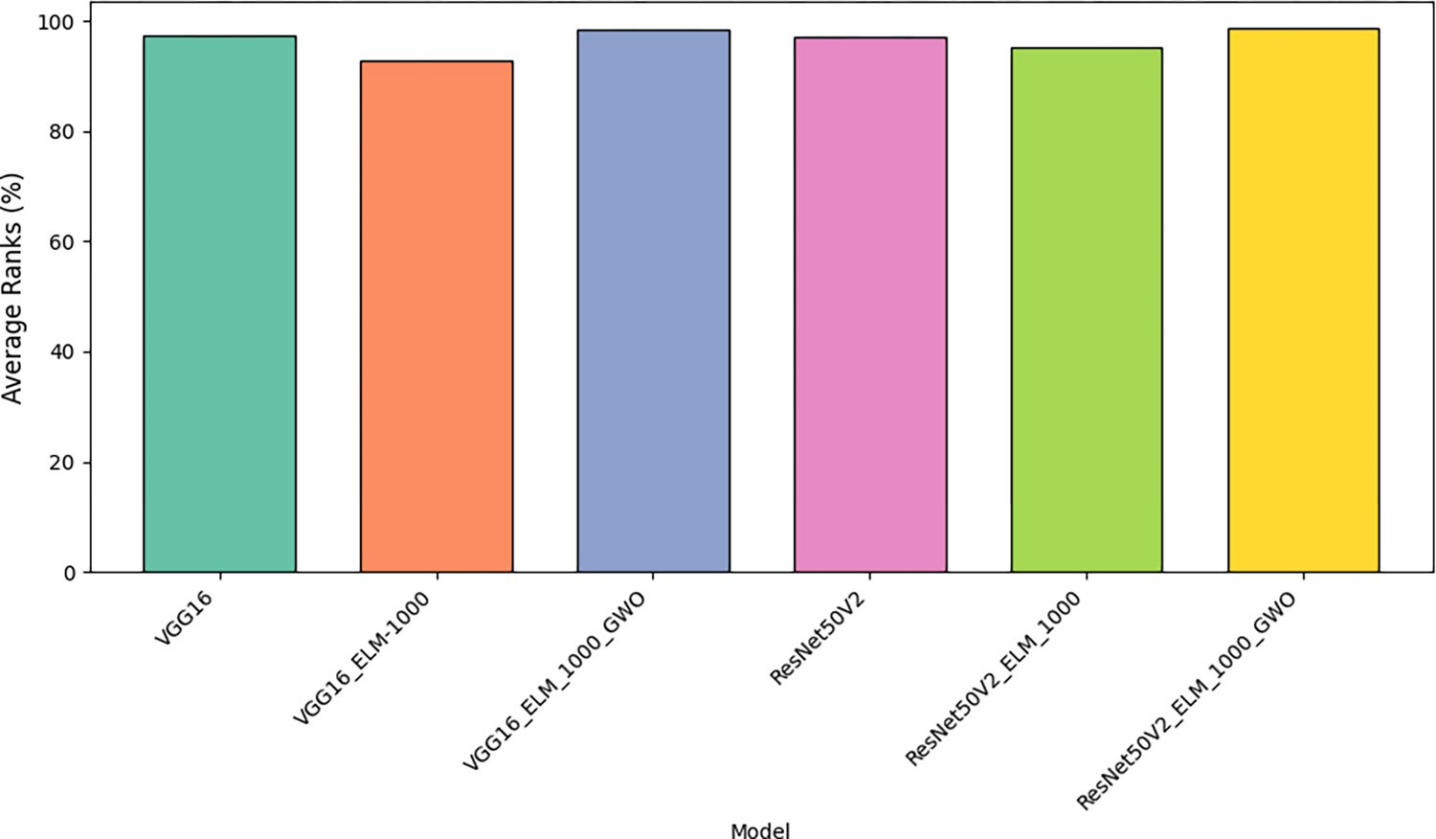
Comparison of average measurement criteria among models.

**Fig 18:**
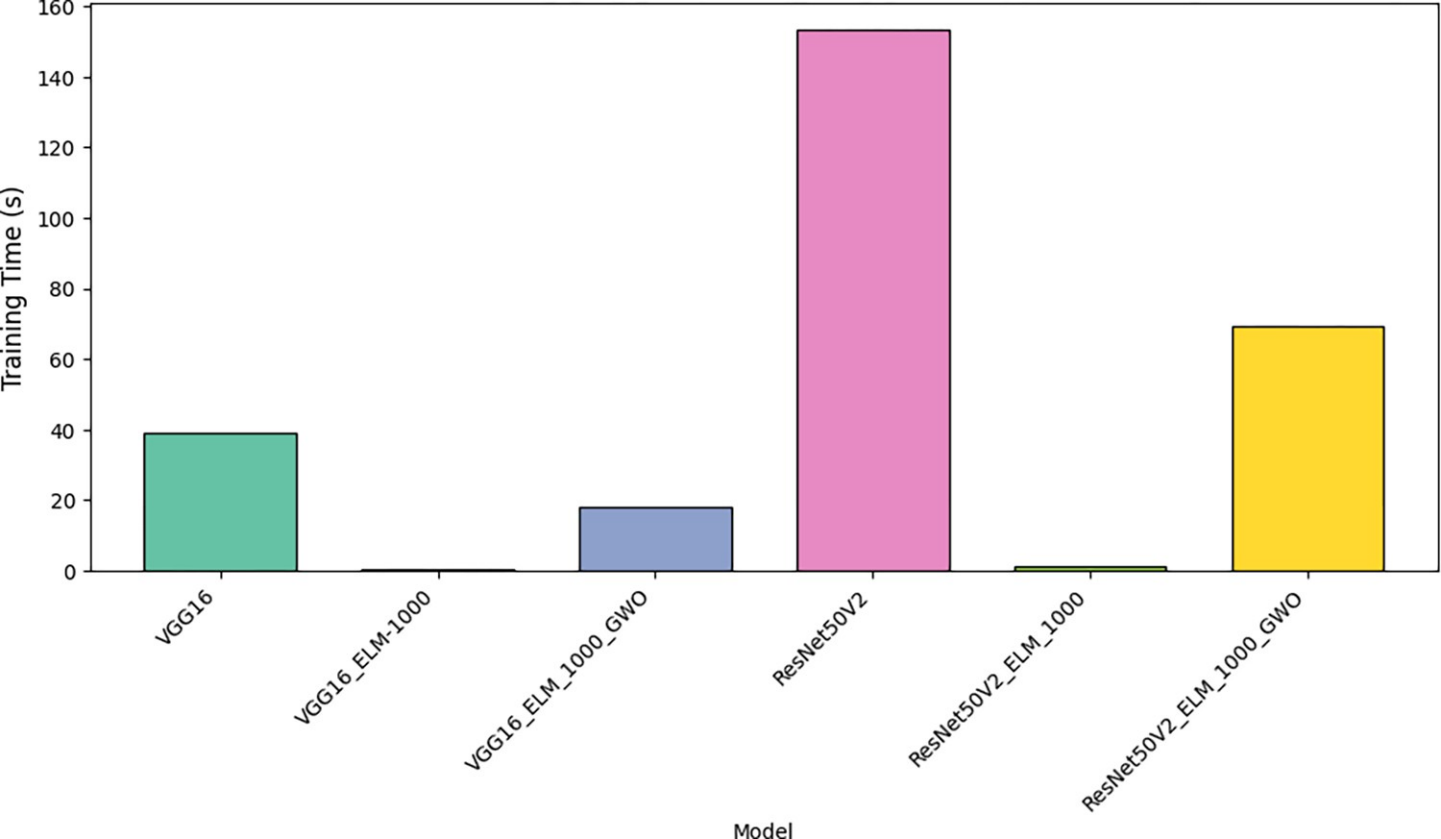
Comparative analysis of training time for various DL models.

The performance outcomes of the proposed DCNNs were obtained using Google Collab shared hardware a T4 graphics card, and is presented in Tables 4 and 5, as well as Figs 10–14. The performance results of the networks shown in Tables 6–8 and Figs 15–18 were acquired utilizing shared hardware and CPU resources provided by Google Collab. Due to this rationale, the values obtained for the table’s shared parameters exhibit variation.

**Table 6:** The statistical results for all experiments of the DCNN_ELMs based on 25 independent runs.

Results of the mean						
Model	Mean of the sensitivity (%)	Mean of the specificity (%)	Mean of the precision (%)	Mean of the F1 Score (%)	Mean of the accuracy (%)	Mean of the MCC (%)
VGG16_ELM-4000	97.7619	98.8237	96.9394	97.9781	97.8461	97.6260
VGG16_ELM-1000	91.5144	96.9638	92.8416	91.6290	91.6921	91.3280
ResNet50V2_ELM_4000	97.1349	98.6523	97.6855	97.3091	96.9230	96.6945
ResNet50V2_ELM_1000	94.8809	97.8789	95.3290	94.7338	94.1538	93.9879
Results of the STD						
Model	STD of the sensitivity (%)	STD of the specificity (%)	STD of the precision (%)	STD of the F1 Score (%)	STD of the accuracy (%)	STD of the MCC (%)
VGG16_ELM-4000	1.5547	1.2325	1.1739	1.4529	1.5689	1.6235
VGG16_ELM-1000	3.5110	2.1234	2.6877	3.3134	3.0147	3.1569
ResNet50V2_ELM_4000	1.9763	1.6314	1.8438	1.9206	2.1757	2.3845
ResNet50V2_ELM_1000	2.8134	2.4695	2.9853	3.1201	3.7684	3.9867
Results of the RMSE						
Model	RMSE of the sensitivity (%)	RMSE of the specificity (%)	RMSE of the precision (%)	RMSE of the F1 Score (%)	RMSE of the accuracy (%)	RMSE of the MCC (%)
VGG16_ELM-4000	2.7251	1.0256	1.9539	2.4897	2.6646	2.8312
VGG16_ELM-1000	9.1832	6.8274	7.6463	9.0029	8.8377	9.2128
ResNet50V2_ELM_4000	3.4806	2.1593	2.9590	3.3059	3.7684	3.8765
ResNet50V2_ELM_1000	5.8412	5.1232	5.5434	6.1210	6.7762	6.8596

**Table 7:** Comparative performance analysis of hybrid DL models using VGG16 and ResNet50V2 with ELM and GWO integration.

Results of the Training Time in 5-fold								
k	VGG16	VGG16_ELM_1000	VGG16_ELM_4000	VGG16_ELM_1000_GWO	ResNet50V2	Resnet50v2_ELM_1000	resnet50v2_ELM_4000	Resnet50v2_ELM_1000_GWO
1	39.0408	0.3532	1.3897	18.1567	152.9859	1.3639	6.1361	68.7228
2	38.8797	0.3532	1.3693	18.1194	153.0510	1.3853	5.3686	69.2732
3	39.0265	0.3536	1.3799	18.0744	153.3002	1.3748	5.7518	69.2416
4	38.9293	0.3529	1.3790	18.0492	153.5842	1.3865	5.3507	69.7603
5	39.2635	0.3562	1.3771	18.3896	153.4103	1.3545	5.3232	69.7578
mean	39.0279	0.3538	1.379	18.1578	153.2663	1.3730	5.5860	69.3511
Results of the Accuracy (%) in 5-fold								
k	VGG16	VGG16_ELM_1000	VGG16_ELM_4000	VGG16_ELM_1000_GWO	ResNet50V2	Resnet50v2_ELM_1000	resnet50v2_ELM_4000	Resnet50v2_ELM_1000_GWO
1	96.9230	90.7692	98.4615	99.9230	98.4615	93.8461	98.4615	98.3846
2	98.4615	92.3076	95.3846	98.9230	95.3846	100	93.8461	97.4615
3	96.9230	96.9230	96.9230	99.4615	95.3846	89.2307	96.9230	97.3846
4	98.4615	87.6923	98.4615	97.3076	96.9230	93.8461	95.3846	97.7692
5	96.9230	90.7692	100	98.3846	98.4615	93.8461	100	96.9230
mean	97.5384	91.6922	97.8461	98.8059	96.9230	94.1538	96.9221	97.5845
Results of the Precisions (%) in 5-fold								
k	VGG16	VGG16_ELM_1000	VGG16_ELM_4000	VGG16_ELM_1000_GWO	ResNet50V2	Resnet50v2_ELM_1000	resnet50v2_ELM_4000	Resnet50v2_ELM_1000_GWO
1	97.9166	90.5820	98.8888	99.9166	98.8888	96.2962	98.88888	98.0396
2	98.8888	93.6111	96.4957	99.2222	95.9054	100	94.5634	98.8888
3	97.9166	97.5274	97.9166	98.7179	96.1385	90.7305	97.9166	97.1111
4	98.8888	89.9478	98.8888	98.4117	97.5274	94.5634	97.0588	97.8030
5	97.5274	92.5396	100	98.4285	98.8888	95.0549	100	98.9166
mean	98.2276	92.8415	98.4379	98.9393	97.4697	95.329	97.6855	98.1518
Results of the Sensitivity (%) in 5-fold								
k	VGG16	VGG16_ELM_1000	VGG16_ELM_4000	VGG16_ELM_1000_GWO	ResNet50V2	Resnet50v2_ELM_1000	resnet50v2_ELM_4000	Resnet50v2_ELM_1000_GWO
1	96.6666	91.2301	98.3333	98.9444	98.6111	93.6111	98.6111	98.7539
2	98.3333	93.3730	95.4761	98.4444	96.0317	100	94.5634	99.6111
3	96.9444	97.1428	96.6666	99.8095	95.9054	91.4682	96.9444	99.7539
4	98.3333	88.4054	98.3333	98.8795	97.1428	94.7619	95.5555	98.7857
5	97.1428	87.4206	100	97.3650	98.3333	94.5634	100	98.7269
mean	97.4840	91.5143	97.7618	98.6885	97.2048	94.8809	97.1348	99.1263
Results of the F1_scores (%) in 5-fold								
k	VGG16	VGG16_ELM_1000	VGG16_ELM_4000	VGG16_ELM_1000_GWO	ResNet50V2	Resnet50v2_ELM_1000	resnet50v2_ELM_4000	Resnet50v2_ELM_1000_GWO
1	97.0370	90.7337	98.5480	99.2870	98.7006	94.2508	98.7006	99.2613
2	98.5480	93.1927	95.7573	97.1515	95.9023	100	94.5634	98.7006
3	97.2870	97.2656	97.0370	98.7160	95.9603	90.1895	97.2870	98.8845
4	98.5480	88.4633	98.5480	98.0896	97.2656	94.4871	95.9947	98.9047
5	97.2656	88.4895	100	98.1351	98.5480	94.7418	100	99.3737
mean	97.7371	91.6289	97.9780	98.3792	97.2753	94.7338	97.3091	99.0249
Results of the MCC (%) in 5-fold								
k	VGG16	VGG16_ELM_1000	VGG16_ELM_4000	VGG16_ELM_1000_GWO	ResNet50V2	Resnet50v2_ELM_1000	resnet50v2_ELM_4000	Resnet50v2_ELM_1000_GWO
1	96.0121	90.4527	98.2294	97.5974	95.9281	91.5882	95.4611	99.8364
2	96.1554	92.3854	99.1534	98.4080	97.3707	94.9187	96.2147	98.5374
3	95.0975	90.9673	96.6177	98.6475	97.0389	92.8138	99.2957	99.4651
4	95.8200	94.3528	96.9632	96.5618	95.4623	94.7183	96.2463	97.3485
5	95.7108	90.4816	97.1660	99.4476	97.0807	95.9002	96.2545	99.9293
mean	95.7791	91.7279	97.6259	98.0964	96.5761	93.9878	96.6944	99.0233
Results of the Specificity (%) in 5-fold								
k	VGG16	VGG16_ELM_1000	VGG16_ELM_4000	VGG16_ELM_1000_GWO	ResNet50V2	Resnet50v2_ELM_1000	resnet50v2_ELM_4000	Resnet50v2_ELM_1000_GWO
1	97.7203	95.5848	99.0185	98.7285	97.0056	98.4638	99.8554	98.7054
2	95.3082	97.9129	98.6243	98.9069	97.1809	97.9582	97.2644	98.5199
3	98.0914	95.6467	97.9171	99.6403	97.9931	96.7923	98.0648	99.7470
4	99.6209	98.7566	100	99.2436	99.4074	98.1254	100	100
5	97.0815	96.9177	98.4883	98.3649	99.0298	98.0546	97.6659	98.2980
mean	97.5644	96.9638	98.8096	98.9768	98.1233	97.8789	98.5701	99.0540

**Table 8:** Complexity analysis of various models.

R	Model	Training time (s)	Number of network parameters (million)	FLOPS (million)	Average Ranks (%)
1	VGG16	**39.0279**	25.8	51	**97.3884**
2	VGG16_ELM_1000	**0.3538**	25	50	**92.7314**
3	VGG16_ELM_GWO	**18.1578**	25	50	**98.4096**
4	ResNet50V2	**153.2663**	102.9	206	**97.2620**
5	ResNet50V2_ELM_1000	**1.3730**	100.3	200	**95.1607**
6	ResNet50V2_ELM_GWO	**69.3511**	100.3	200	**98.6608**

Table 6 presents a comparative analysis of classification results obtained from the two proposed approaches, VGG16-ELM and ResNet50V2-ELM. Results are the mean, standard deviation (STD) and root mean square error (RMSE) percentage, computed based on 25 independent runs. The experiments utilized an ELM hidden layer configured with 4000 and 1000 nodes.

The mean, RMSE, and STD were calculated using the data from 25 runs of each experiment. These statistical measures were used to evaluate the performance of the proposed DVNN-ELM technique in diagnosing PRIM recognition. The three assessments mentioned are widely recognized as the most prevalent statistical evaluation measures [78–80]. The mean quantifies the proximity of the classifier’s overall performance across multiple runs to the best answer. In contrast, the RMSE quantifies the concentration of the results from various runs around the perfect solution. The STD quantifies the extent to which the outcomes of multiple trials deviate from the average [78–80].

In the present study, a high mean value close to 100.00% indicates that the classifier performed well in various runs. Similarly, a low value for RMSE and STD suggests that the classifier consistently created results similar to or almost equal to 100.00%. The statistical findings for all experiments of the proposed DCNN-ELM techniques are presented in Table 6. Eqs **21**–**23** are utilized for the computation of the μ, RMSE, and STD [78, 80].


$$\mu =\frac{\sum_{i=1}^{N}X_{i}}{N}$$ (21)

$$RMSE=\sqrt{\frac{\sum_{i=1}^{N}{\left(X_{i}-O\right)}^{2}}{N}}$$ (22)

$$SDT=\sqrt{\frac{\sum_{i=1}^{N}{\left(X_{i}-\mu \right)}^{2}}{N}}$$ (23)

The symbol μ indicates the population means, *X*_*i*_ represents each value in the population, N represents the total number of values, and O represents the observed or optimal value, which is 100.00%.

Based on the findings in Table 6, the mean values of all measurements are a close to 100.00%. This indicates that the DCNN_ELM algorithms consistently produced high levels of accuracy, precision, recall, F1-score, sensitivity, MCC, and specificity in the majority of the 25 runs. The low values of RMSE and STD, demonstrate the usefulness of the DCNN-ELM method in obtaining a high classification performance during the 25 runs.

Table 6 shows the VGG16_ELM-4000 model demonstrates exceptional performance, achieving the best mean sensitivity (97.76%), specificity (98.82%), precision (96.93%), F1 score (97.97%), accuracy (97.84%), and MCC (97.62%). These findings indicate that the model demonstrates a robust equilibrium across all assessed metrics, rendering it highly dependable for classification tasks. VGG16_ELM-1000 exhibits inferior performance in comparison to the 4000 feature set, as evidenced by lower mean values of sensitivity (91.51%), specificity (96.96%), precision (92.84%), F1 score (91.62%), accuracy (91.69%), and MCC (91.32%). The decrease in the size of the feature set may impact the performance, but it still has a commendable classification capability. ResNet50V2_ELM_4000 performs somewhat worse than VGG16_ELM-4000 but surpasses the ResNet variation with reduced features. The model attains a mean sensitivity of 97.13%, specificity of 98.65%, precision of 97.68%, F1 score of 97.30%, accuracy of 96.92%, and MCC of 96.69%. The ResNet50V2_ELM_1000 model exhibits the lowest mean values compared to the other three models while still demonstrating exceptional performance in terms of sensitivity (94.88%), specificity (97.87%), precision (95.32%), F1 score (94.73%), accuracy (94.15%), and MCC (93.98%).

The standard deviation numbers reflect the extent of variation in model performance:

The VGG16_ELM-4000 model exhibits minimal variability, as seen by its low standard deviation (STD) values. This suggests that the model’s performance remains stable across multiple runs. VGG16_ELM-1000 and ResNet50V2_ELM_1000 demonstrate larger traditional deviation values than their 4000-feature equivalents, suggesting a lower level of performance consistency. This could be attributed to the decreased intricacy of the feature space or the model’s susceptibility to the subtle variations in the dataset. Overall, when the number of features reduces from 4000 to 1000, both the VGG16 and ResNet50V2 models exhibit an increase in performance variability, as indicated by more significant standard deviation (STD) values.

The VGG16_ELM-4000 consistently achieves low RMSE values, which confirms its vital dependability and precision in classification tasks In contrast, VGG16_ELM-1000 exhibits the most excellent RMSE values, indicating more significant inaccuracies in the performance measurements. Both ResNet50V2_ELM_4000 and ResNet50V2_ELM_1000 exhibit moderate RMSE values. However, the latter has larger values, suggesting a more significant average error.

To summarize, the models with a more extensive feature set of 4000 show better average performance and have reduced variability and error rates, making them more resilient and dependable. The models with a reduced feature set (1000) provide satisfactory average performance but with more significant variability and error, suggesting they may be more susceptible to the dataset or require more precise adjustments to get ideal performance. Overall, the VGG16_ELM-4000 model demonstrates exceptional stability and accuracy across all criteria.

Nevertheless, when the critical data is divided into three sets, the number of samples available for training the model is considerably diminished, and the results can occasionally be influenced by a random selection of the (train, validation) sets. Cross-validation (CV) is a method that addresses this issue by merely using the test set for the final evaluation without needing the validation set. Practitioners widely employ the K-fold cross-validation (KCV) technique to pick models and estimate errors of classifiers. KCV involves dividing a dataset into k subsets. Some of these subsets are used for model training, while the remaining subsets are used for performance evaluation [81, 82].

Given the utilization of an unbalanced and limited data set in this study, it is recommended to employ the 5-fold cross-validation technique to evaluate the final proposed solutions. The results are presented in the Table 7 and Fig 15.

Fig 16 illustrates the comparative performance of several approaches, namely VGG16, VGG16_ELM, VGG16_ELM_GWO, ResNet50V2, ResNet50V2_ELM, and ResNet50V2_ELM_GWO, evaluating their effectiveness in achieving the desired outcomes.

Fig 17 presents a comparative analysis of the average rank, a specific measurement criterion, across six distinct models: VGG16, VGG16_ELM, VGG16_ELM_GWO, ResNet50V2, ResNet50V2_ELM, and ResNet50V2_ELM_GWO, providing insights into their respective performances.

Table 7 and Figs 15–17 has examined and contrasted the efficacy of traditional deep networks VGG16 and ResNet50V2, along with their combined variants utilizing ELM and GWO methods. This paper employed a 5-fold CV methodology for evaluation, resulting in the subsequent outcomes:

- The training time for the VGG16 and ResNet50V2 models is considerably longer, averaging 39.0279 and 153.2663 seconds, respectively. Conversely, the incorporation of ELM layers significantly decreases the duration of training. VGG16_ELM_1000 and ResNet50v2_ELM_1000, using a smaller feature set of 1000, exhibit mean training times of 0.3538 and 1.3730 seconds, respectively. These times are ten times faster than their non-ELM counterparts. Increasing the feature set to 4000 in VGG16_ELM_4000 and resnet50v2_ELM_4000 leads to a slight increase in time, resulting in average durations of 1.3790 and 5.5860 seconds, respectively. The inclusion of GWO optimization significantly impacts the duration of the training, namely with VGG16_ELM_1000_GWO and Resnet50v2_ELM_1000_GWO. The training length increased significantly to 18.1578 and 69.3511 seconds, respectively, indicating that the optimization step contributes to the computational cost.
- The standard VGG16 model achieves the highest average accuracy of 97.5384%, closely followed by VGG16_ELM_4000 and VGG16_ELM_1000_GWO, which have average accuracies of 97.8461% and 98.8059%, respectively. The findings indicate that utilizing ELM layers and GWO modification can improve the performance of VGG16. ResNet50V2 and its variations demonstrate marginally lower average accuracies, with Resnet50v2_ELM_1000_GWO obtaining an average of 97.5845%.
- Precision is a measure that indicates the proportion of accurate optimistic predictions out of all the optimistic forecasts made. The VGG16_ELM_1000_GWO model demonstrated exceptional performance with a mean precision of 98.9393%, which signifies its dependability in accurately identifying negative cases as unfavorable. The precision of Resnet50v2_ELM_1000_GWO closely aligns with a mean of 98.1518%, indicating that GWO optimization improves precision for both architectures.
- The VGG16_ELM_1000_GWO model exhibits exceptional sensitivity, with an average value of 98.6885%. The Resnet50v2_ELM_1000_GWO model shows a notable sensitivity, with an average of 99.1263%, indicating that the GWO optimization enhances the models’ capacity to identify positive cases in many scenarios.
- The two GWO-optimized models, VGG16_ELM_1000_GWO and Resnet50v2_ELM_1000_GWO, exhibit strong performance in terms of accuracy and sensitivity, as seen by their high mean F1 scores of 98.3792% and 99.0249%, respectively.
- The models VGG16_ELM_1000_GWO and Resnet50v2_ELM_1000_GWO demonstrate impressive MCC of 98.0964% and 99.0233%, respectively, indicating their excellent predictive accuracy and ability to handle class imbalances effectively.
- The Resnet50v2_ELM_1000_GWO model demonstrates the highest mean specificity of 99.0540%, suggesting its extraordinary capability to identify and reject non-PRI cases accurately. This is particularly important in applications where false alarms might incur significant costs.

Overall, utilizing VGG16 and ResNet50V2 models with ELM layers and GWO optimization has exhibited noteworthy enhancements in both efficiency and efficacy. They significantly decrease the duration of training sessions while improving all performance measures, such as accuracy, precision, sensitivity, F1 scores, MCC, and specificity. The GWO-optimized variations achieve a commendable equilibrium between the time taken for training and the performance in classification. This makes them well-suited for PRI classification tasks in real-world scenarios when accuracy and efficiency are paramount. The uniformity in performance across all folds suggests that the models are robust and capable of effectively adapting to unfamiliar input, which is crucial for their use in practical environments. The combination of ELM and GWO optimization demonstrates the promise of these hybrid methodologies in successfully and efficiently addressing complicated classification tasks, as evidenced by the reduced training times and high performance achieved.

Table 8 delineates a comprehensive complexity analysis of several models, specifically VGG16, VGG16_ELM, VGG16_ELM_GWO, ResNet50V2, ResNet50V2_ELM, and ResNet50V2_ELM_GWO. The analysis provides insights into the computational cost, resources required, and the overall complexity of each model.

Fig 18 illustrates the comparative amount of training time required by different DL models including VGG16, VGG16_ELM, VGG16_ELM_GWO, ResNet50V2, ResNet50V2_ELM, and ResNet50V2_ELM_GWO, offering insights into their computational efficiency and time consumption.

Table 8 and Fig 18 demonstrate that the ResNet50V2 models exhibit a considerably higher level of complexity. With FLOPS of over 200 million and parameters of roughly 100 million, these models require the highest amount of computational resources. In contrast, VGG16`models have a shallow level of complexity, with FLOPS of approximately 50 million and around 25 million parameters.

The VGG16_ELM_1000 model distinguishes itself by having an impressively brief training duration of around 0.354 seconds, rendering it the most efficient. Conversely, `ResNet50V2`necessitates the most extended training duration, amounting to 153.266 seconds.

The ResNet50V2_ELM_GWO model has superior performance, achieving a remarkable accuracy of 98.66%. This represents a compromise between the level of computing difficulty and precision since higher performance necessitates more excellent computational resources. Conversely, the model `VGG16_ELM_1000`has the lowest accuracy of 92.73%, indicating that it is a more cost-effective but less effective model.

The analysis of Tables 6–8 and Figs 15–18 indicates a significant decrease in training durations with the incorporation of ELM layers. For example, VGG16_ELM_1000 and ResNet50v2_ELM_1000, which have a reduced feature set of 1000, show training times that is ten times faster than their regular versions. Nevertheless, the incorporation of GWO results in a substantial increase in training duration, which suggests additional computational expenses.

The typical VGG16 model demonstrates exceptional performance with an impressive average accuracy of 97.5384%.The performance of this model is improved by incorporating ELM layers and utilizing GWO optimization. Notably, VGG16_ELM_4000 and VGG16_ELM_1000_GWO have achieved accuracies as high as 98.8059%. The ResNet50V2 models, although exhibiting a somewhat lower average accuracy, also demonstrate enhancements with these alterations.

The VGG16_ELM_1000_GWO model exhibits extraordinarily high precision, a crucial indicator of accurate optimistic predictions. This high precision underscores the model’s trustworthiness. The GWO-optimized models provide exceptional sensitivity and specificity, essential for precisely recognizing positive cases and correctly rejecting non-PRI instances, respectively.

The F1 scores and Matthew’s Correlation Coefficient (MCC) highlight these algorithms’ exceptional prediction accuracy and capability in addressing class imbalances. The Resnet50v2_ELM_1000_GWO model demonstrates the best average specificity, which is particularly important for applications requiring minimal false alarms.

When examining the intricacy of these models, ResNet50V2 distinguishes itself due to its substantial demand on computational resources, as indicated by its FLOPS (floating point operations per second) and parameters. In contrast, VGG16 models exhibit lower complexity while simultaneously demonstrating lower efficiency in terms of accuracy.

Incorporating ELM and GWO optimization into VGG16 and ResNet50V2 models represents notable progress in PRI modulation detection. These models decrease the time required for training and improve performance across different measurements, achieving a harmonious combination of training length and classification effectiveness. The equilibrium is crucial for practical scenarios when precision and efficacy are paramount. The consistent performance observed in all folds indicates the resilience and flexibility of these hybrid techniques, underscoring their potential to tackle intricate classification issues.

## 5. Discussion

Given the provided data, it can be inferred that a clear trade-off exists between model performance and computational complexity across the different DCNN models explored. ResNet50V2 stands out with the highest scores in most metrics, emphasizing its proficiency in classification tasks but at a substantial computational cost. It shows increased FLOPS and network parameters, indicating potentially higher resource demands and longer inference times. EfficientNetB0, while having competitive complexity metrics, lags significantly in performance, suggesting a need for a balanced approach between efficiency and efficacy. Models like VGG16 and DenseNet121 exemplify lower computational demands and faster training, making them feasible and adaptable to various applications, notably when resources are constrained. Moreover, ELM-enhanced models such as VGG16 ELM-4000 show significant improvements in efficiency and performance, albeit with increased training times in some instances like ResNet50V2_ELM_4000. GWO-enhanced models, despite their superior performance in classification metrics, are considerably resource-intensive, emphasizing the necessity for optimizations and strategic model selections based on task-specific requirements and constraints. In essence, choosing the suitable model necessitates meticulously considering the balance between performance, computational efficiency, and resource availability tailored to the distinct needs of each classification task.

## 6. Conclusion

This work is the inaugural utilization of PRI sound for PRIM recognition. This study presents an innovative three-phase methodology for identifying the six prevalent kinds of PRIM. The initial step of this methodology was training a DCNN based on transfer learning, which served as a feature extractor. Subsequently, an ELM was substituted for the final fully connected layers to enhance the proposed model’s time complexity. Later, the introduction of GWO aimed to mitigate the space complexity associated with the proposed paradigm. This research also presents a novel experimental dataset of PRI patterns specifically tailored for recognition measurement, marking its inaugural introduction. This study incorporates eight pre-trained convolutional neural network models, including the VGG and the ResNet. The models have undergone satisfactory testing and evaluation using the PRI sound image dataset. The implemented classifiers’ outcomes showed that VGG16 and ResNet50V2 models obtained the best recognition accuracy with values of 97.53% and training time of 39.02 seconds and 96.92% and training time of 153.26 seconds, respectively. These values increased to 98.80%, a training time of 18.15 seconds, and a 97.58 training time of 69.35 seconds with the evolution of these networks with ELM and GWO, respectively. When evaluating all six measurement criteria, ResNet50V2_ELM_GWO is given the highest rating, while VGG 16_ELM_GWO is given the second-highest score.

For future research endeavors, several suggestions can be put forward. Optimizing prevailing models like ResNet50V2 and VGG16 warrants further exploration as it promises benefits in reducing training time and computational complexity. A deeper and more comprehensive investigation into current methodologies available for trimming model complexity will likely bear fruitful outcomes. Furthermore, scrutinizing other models, including EfficientNet and diverse versions of MobileNet, is conducive to discovering more efficacious and economical models. Experimentation with novel and contemporary techniques in deep learning, such as Transfer Learning and Meta-Learning, is poised to elevate the performance of models. Lastly, the adoption of advanced and potent hardware has the potential to abbreviate the duration of training times significantly.

## References

[1] Feng H.C., Tang B., and Wan T., Radar pulse repetition interval modulation recognition with combined net and domain-adaptive few-shot learning. Digital Signal Processing, 2022. 127: p. 103562.

[2] Dadgarnia A. and Sadeghi M.T., Automatic recognition of pulse repetition interval modulation using temporal convolutional network. IET Signal Processing, 2021. 15(9): p. 633–648.

[3] Han J.-W. and Park C.H., A unified method for deinterleaving and PRI modulation recognition of radar pulses based on deep neural networks. IEEE Access, 2021. 9: p. 89360–89375.

[4] Qiao G., et al., Recognition and parameter estimation of spaceborne synthetic aperture radar pulse repetition interval modulation based on short time modified pulse repetition interval transform. IET Radar, Sonar & Navigation, 2022. 16(10): p. 1696–1716.

[5] Wang J., et al., A Radar Emitter Recognition Mechanism Based on IFS-Tri-Training Classification Processing. Electronics, 2022. 11(7): p. 1078.

[6] Wiley R., ELINT: The interception and analysis of radar signals. 2006: Artech.

[7] Zhang, D., et al. Distributed Radar PRI Sequence Classification using K-medoids Algorithm and Feedforward Neural Networks. in 2021 IEEE 5th Information Technology, Networking, Electronic and Automation Control Conference (ITNEC). 2021. IEEE.

[8] Svensson A., Classification of Radar Emitters Based on Pulse Repetition Interval using Machine Learning. 2022.

[9] Nguyen H.P., Nguyen H.Q., and Ngo D.T. Deep Learning for Pulse Repetition Interval Classification. in ICPRAM. 2019.

[10] Huang C.-Q., et al., Dual-graph attention convolution network for 3-D point cloud classification. IEEE Transactions on Neural Networks and Learning Systems, 2022. 10.1109/TNNLS.2022.3162301 35385393

[11] Huang G.-B., Wang D.H., and Lan Y., Extreme learning machines: a survey. International journal of machine learning and cybernetics, 2011. 2(2): p. 107–122.

[12] Albadr, M.A.A., et al. Extreme learning machine for automatic language identification utilizing emotion speech data. in 2021 international conference on electrical, communication, and computer engineering (ICECCE). 2021. IEEE.

[13] Albadr M.A.A. and Tiun S., Spoken language identification based on particle swarm optimisation–extreme learning machine approach. Circuits, Systems, and Signal Processing, 2020. 39: p. 4596–4622.

[14] Xu Y., et al., Real-time transient stability assessment model using extreme learning machine. IET generation, transmission & distribution, 2011. 5(3): p. 314–322.

[15] Zhou Z., et al., Color difference classification of solid color printing and dyeing products based on optimization of the extreme learning machine of the improved whale optimization algorithm. Textile Research Journal, 2020. 90(2): p. 135–155.

[16] Chen H., et al., An enhanced Bacterial Foraging Optimization and its application for training kernel extreme learning machine. Applied Soft Computing, 2020. 86: p. 105884.

[17] Albadr M.A.A., et al., Speech emotion recognition using optimized genetic algorithm-extreme learning machine. Multimedia Tools and Applications, 2022. 81(17): p. 23963–23989.

[18] Tian Q., et al., Real-time human cross-race aging-related face appearance detection with deep convolution architecture. Journal of Real-Time Image Processing, 2020. 17(1): p. 83–93.

[19] Haut J.M., et al., Fast dimensionality reduction and classification of hyperspectral images with extreme learning machines. Journal of Real-Time Image Processing, 2018. 15(3): p. 439–462.

[20] Zhao, G., et al. On improving the conditioning of extreme learning machine: a linear case. in 2009 7th International Conference on Information, Communications and Signal Processing (ICICS). 2009. IEEE.

[21] Albadr M.A.A., et al., Grey wolf optimization-extreme learning machine for automatic spoken language identification. Multimedia Tools and Applications, 2023: p. 1–27.

[22] Mirjalili S., Mirjalili S.M., and Lewis A., Grey wolf optimizer. Advances in engineering software, 2014. 69: p. 46–61.

[23] Faris H., et al., Grey wolf optimizer: a review of recent variants and applications. Neural computing and applications, 2018. 30: p. 413–435.

[24] Ranney K. and Tom K., A Survey of Methods for Estimating Pulse Width and Pulse Repetition Interval. 2020.

[25] Xie L., et al., Self-feature-based point cloud registration method with a novel convolutional Siamese point net for optical measurement of blade profile. Mechanical Systems and Signal Processing, 2022. 178: p. 109243.

[26] Duan J., et al., Fixed-time time-varying output formation–containment control of heterogeneous general multi-agent systems. ISA transactions, 2023. 137: p. 210–221. doi: 10.1016/j.isatra.2023.01.008 36653249

[27] Kumar N.U., Dhananjayulu V., and Kumar V.A., Deinterleaving of radar signals and its parameter estimation in EW environment. International Journal of Emerging Technology and Advanced Engineering, 2014. 4(9): p. 490–494.

[28] Sridharan S., et al., Improved pulse repetition interval (PRI) deinterleaving for electronic support measure (ESM) receiver. Int. Journal of Advanced Computing and Electronics Technology (IJACET), 2015. 2(3): p. 37–43.

[29] Ata’a A. and Abdullah S., Deinterleaving of radar signals and PRF identification algorithms. IET radar, sonar & navigation, 2007. 1(5): p. 340–347.

[30] Bagheri M. and Sedaaghi M.H., A new approach to pulse deinterleaving based on adaptive thresholding. Turkish Journal of Electrical Engineering and Computer Sciences, 2017. 25(5): p. 3827–3838.

[31] Liu, J., H. Meng, and X. Wang. A new pulse deinterleaving algorithm based on multiple hypothesis tracking. in 2009 International Radar Conference" Surveillance for a Safer World"(RADAR 2009). 2009. IEEE.

[32] Ryoo Y.-J., Song K.-H., and Kim W.-W., Recognition of PRI modulation types of radar signals using the autocorrelation. IEICE transactions on communications, 2007. 90(5): p. 1290–1294.

[33] Ahmadi M. and Mohamedpour K., PRI modulation type recognition using level clustering and autocorrelation. American Journal of Signal Processing, 2012. 2(5): p. 83–91.

[34] Kauppi, J.-P. and K. Martikainen. An efficient set of features for pulse repetition interval modulation recognition. in 2007 IET International Conference on Radar Systems. 2007. IET.

[35] Ghani, K.A., et al. Pulse repetition interval analysis using decimated Walsh-Hadamard transform. in 2017 IEEE Radar Conference (RadarConf). 2017. IEEE.

[36] Ahmed, U.I., et al. Robust pulse repetition interval (PRI) classification scheme under complex multi emitter scenario. in 2018 22nd International Microwave and Radar Conference (MIKON). 2018. IEEE.

[37] Liu, Y. and Q. Zhang. An improved algorithm for PRI modulation recognition. in 2017 IEEE International Conference on Signal Processing, Communications and Computing (ICSPCC). 2017. IEEE.

[38] Keshavarzi, M., A.M. Pezeshk, and F. Farzaneh. A new method for detection of complex pulse repetition interval modulations. in 2012 IEEE 11th International Conference on Signal Processing. 2012. IEEE.

[39] Song, K.-H., et al. Pulse repetition interval modulation recognition using symbolization. in 2010 International Conference on Digital Image Computing: Techniques and Applications. 2010. IEEE.

[40] Hu, G. and Y. Liu. An efficient method of pulse repetition interval modulation recognition. in 2010 International Conference on Communications and Mobile Computing. 2010. IEEE.

[41] Noone G.P. A neural approach to automatic pulse repetition interval modulation recognition. in 1999 Information, Decision and Control. Data and Information Fusion Symposium, Signal Processing and Communications Symposium and Decision and Control Symposium. Proceedings (Cat. No. 99EX251). 1999. IEEE.

[42] Heaton, J., Ian Goodfellow, Yoshua Bengio, and Aaron Courville: Deep learning: The MIT Press, 2016, 800 pp, ISBN: 0262035618. Genetic programming and evolvable machines, 2018. 19(1–2): p. 305–307.

[43] LeCun Y., Bengio Y., and Hinton G., Deep learning. nature, 2015. 521(7553): p. 436–444. 10.1038/nature14539 26017442

[44] Li D., Ortegas K.D., and White M., Exploring the Computational Effects of Advanced Deep Neural Networks on Logical and Activity Learning for Enhanced Thinking Skills. Systems, 2023. 11(7): p. 319.

[45] Chen X., et al., 3d object proposals for accurate object class detection. Advances in neural information processing systems, 2015. 28.

[46] Li X., Liu Z., and Huang Z., Attention-based radar PRI modulation recognition with recurrent neural networks. IEEE Access, 2020. 8: p. 57426–57436.

[47] Liu F., Zhang G., and Lu J., Multisource heterogeneous unsupervised domain adaptation via fuzzy relation neural networks. IEEE Transactions on Fuzzy Systems, 2020. 29(11): p. 3308–3322.

[48] Zhang K., et al., Training effective deep reinforcement learning agents for real-time life-cycle production optimization. Journal of Petroleum Science and Engineering, 2022. 208: p. 109766.

[49] Li B., et al., A distributionally robust optimization based method for stochastic model predictive control. IEEE Transactions on Automatic Control, 2021. 67(11): p. 5762–5776.

[50] Li X., et al., Toward convolutional neural networks on pulse repetition interval modulation recognition. IEEE Communications Letters, 2018. 22(11): p. 2286–2289.

[51] Hekrdla, M. and A. Heřmánek. Deep Convolutional Neural Network Classifier of Pulse Repetition Interval Modulations. in 2019 International Radar Conference (RADAR). 2019. IEEE.

[52] O’Shea, K. and R. Nash, An introduction to convolutional neural networks. arXiv preprint arXiv:1511.08458, 2015.

[53] LeCun Y., LeNet-5, convolutional neural networks. URL: http://yann.lecun.com/exdb/lenet, 2015. 20(5): p. 14.

[54] Krizhevsky A., Sutskever I., and Hinton G.E., ImageNet classification with deep convolutional neural networks. Communications of the ACM, 2017. 60(6): p. 84–90.

[55] Zeiler, M.D. and R. Fergus. Visualizing and understanding convolutional networks. in Computer Vision–ECCV 2014: 13th European Conference, Zurich, Switzerland, September 6–12, 2014, Proceedings, Part I 13. 2014. Springer.

[56] Szegedy, C., et al. Going deeper with convolutions. in Proceedings of the IEEE conference on computer vision and pattern recognition. 2015.

[57] Simonyan, K. and A. Zisserman, Very deep convolutional networks for large-scale image recognition. arXiv preprint arXiv:1409.1556, 2014.

[58] Carnagie J.O., et al., Essential oil plants image classification using xception model. Procedia Computer Science, 2022. 204: p. 395–402.

[59] Cheng M.-Y., Sholeh M.N., and Harsono K., Automated vision-based post-earthquake safety assessment for bridges using STF-PointRend and EfficientNetB0. Structural Health Monitoring, 2023: p. 14759217231168709.

[60] de Zarzà I., de Curtò J., and Calafate C.T., Detection of glaucoma using three-stage training with EfficientNet. Intelligent Systems with Applications, 2022. 16: p. 200140.

[61] Yang H., et al., A novel method for peanut variety identification and classification by Improved VGG16. Scientific Reports, 2021. 11(1): p. 15756. doi: 10.1038/s41598-021-95240-y 34344983 PMC8333428

[62] Mpova, L., T.C. Shongwe, and A. Hasan. The Classification and Detection of Cyanosis Images on Lightly and Darkly Pigmented Individual Human Skins Applying Simple CNN and Fine-Tuned VGG16 Models in TensorFlow’s Keras API. in 2023 IEEE International Conference on Computational Intelligence and Virtual Environments for Measurement Systems and Applications (CIVEMSA). 2023. IEEE.

[63] Raje, N.R. and A. Jadhav. Automated Diagnosis of Pneumonia through Capsule Network in conjunction with ResNet50v2 model. in 2022 International Conference on Emerging Smart Computing and Informatics (ESCI). 2022. IEEE.

[64] Haruna U., Ali R., and Man M., A new modification CNN using VGG19 and ResNet50V2 for classification of COVID-19 from X-ray radiograph images. Indonesian Journal of Electrical Engineering and Computer Science, 2023. 31(1): p. 369–377.

[65] Suthar, O., V. Katkar, and K. Vaghela. Person Recognition using Gait Energy Image, MobileNetV3Small and Machine Learning. in 2023 IEEE 3rd International Conference on Technology, Engineering, Management for Societal impact using Marketing, Entrepreneurship and Talent (TEMSMET). 2023. IEEE.

[66] Nandhini S. and Ashokkumar K., An automatic plant leaf disease identification using DenseNet-121 architecture with a mutation-based henry gas solubility optimization algorithm. Neural Computing and Applications, 2022: p. 1–22.

[67] Alwakid G., et al., Deep learning-enhanced diabetic retinopathy image classification. Digital Health, 2023. 9: p. 20552076231194942. doi: 10.1177/20552076231194942 37588156 PMC10426308

[68] Niu Z., et al., The research on 220GHz multicarrier high-speed communication system. China Communications, 2020. 17(3): p. 131–139.

[69] Wang J., et al., A review on extreme learning machine. Multimedia Tools and Applications, 2022. 81(29): p. 41611–41660.

[70] Zhu S., et al., Synchronous measuring of triptolide changes in rat brain and blood and its application to a comparative pharmacokinetic study in normal and Alzheimer’s disease rats. Journal of Pharmaceutical and Biomedical Analysis, 2020. 185: p. 113263. doi: 10.1016/j.jpba.2020.113263 32203895

[71] Liu G., et al., Antibacterial activity and mechanism of bifidocin A against Listeria monocytogenes. Food Control, 2017. 73: p. 854–861.

[72] Li Y., Lin X., and Liu J., An improved gray wolf optimization algorithm to solve engineering problems. Sustainability, 2021. 13(6): p. 3208.

[73] Sainburg T., Thielk M., and Gentner T.Q., Finding, visualizing, and quantifying latent structure across diverse animal vocal repertoires. PLoS computational biology, 2020. 16(10): p. e1008228. doi: 10.1371/journal.pcbi.1008228 33057332 PMC7591061

[74] Chicco D. and Jurman G., The advantages of the Matthews correlation coefficient (MCC) over F1 score and accuracy in binary classification evaluation. BMC genomics, 2020. 21(1): p. 1–13. doi: 10.1186/s12864-019-6413-7 31898477 PMC6941312

[75] Xie X., et al., A simple Monte Carlo method for estimating the chance of a cyclone impact. Natural Hazards, 2021. 107: p. 2573–2582.

[76] Wang J., et al., Control of time delay force feedback teleoperation system with finite time convergence. Frontiers in Neurorobotics, 2022. 16: p. 877069. doi: 10.3389/fnbot.2022.877069 35599666 PMC9120597

[77] Jia D., et al., Lubrication-enhanced mechanisms of titanium alloy grinding using lecithin biolubricant. Tribology International, 2022. 169: p. 107461.

[78] Albadr M.A.A., et al., Breast cancer diagnosis using the fast learning network algorithm. Frontiers in Oncology, 2023. 13: p. 1150840. doi: 10.3389/fonc.2023.1150840 37434975 PMC10332166

[79] Albadr M.A., et al., Genetic algorithm based on natural selection theory for optimization problems. Symmetry, 2020. 12(11): p. 1758.

[80] Albadr M.A.A., et al., Spoken language identification based on optimised genetic algorithm–extreme learning machine approach. International Journal of Speech Technology, 2019. 22: p. 711–727.

[81] Zhang X. and Liu C.-A., Model averaging prediction by K-fold cross-validation. Journal of Econometrics, 2023. 235(1): p. 280–301.

[82] Vu H.L., et al., Analysis of input set characteristics and variances on k-fold cross validation for a Recurrent Neural Network model on waste disposal rate estimation. Journal of environmental management, 2022. 311: p. 114869. doi: 10.1016/j.jenvman.2022.114869 35287077

